# Distribution and Influences on Butterfly Diversity in Urban Park Green Spaces: A Case Study of Harbin, China

**DOI:** 10.1002/ece3.71554

**Published:** 2025-06-12

**Authors:** Kai Wang, Yuxin Qi, Yuandong Hu, Dan Han

**Affiliations:** ^1^ College of Landscape Architecture Northeast Forestry University Harbin China; ^2^ State Key Laboratory of Earth Surface Processes and Resource Ecology Beijing Normal University Beijing China; ^3^ Faculty of Geographical Science Beijing Normal University Beijing China; ^4^ Institute for Interdisciplinary and Innovation Research Xi'an University of Architecture and Technology Xi'an China; ^5^ School of Design and Architecture Zhejiang University of Technology Hangzhou China

**Keywords:** butterfly species richness, landscape matrix, spatial heterogeneity, urban parks, urbanization

## Abstract

With the acceleration of urbanization, urban parks play a critical role in protecting biodiversity and maintaining ecosystem stability. As environmental indicator species, butterflies exhibit high sensitivity to environmental changes and anthropogenic disturbances, making them ideal subjects for studying urbanization's impacts on biodiversity. This study focuses on the effects of spatial heterogeneity in the surrounding matrix and its interaction with park resources on butterfly diversity in urban parks. Using Harbin City in Heilongjiang Province, northeastern China, as the study area, we collected data on butterfly species richness and park characteristics from 44 urban parks. Spatial heterogeneity was quantified through landscape metrics across eight spatial scales (100–2500 m), and generalized linear mixed models were employed to analyze butterfly community responses to park characteristics and landscape patterns (composition and configuration) at multiple spatial scales. The results identified 38 butterfly species and 12,885 individuals in Harbin's urban area, with significantly higher species richness in urban fringe zones compared to the city center. Heterogeneity indicators across different spatial scales showed complex relationships with butterfly communities. Butterfly community structure and composition demonstrated highest sensitivity to landscape heterogeneity at 100 and 200 m scales, with optimal model explanatory power for richness, abundance, and diversity being 67.8%, 81.1%, and 38.8% respectively. Key predictors exhibited hierarchical differentiation: richness was primarily positively driven by flowering plant richness and distance from the city center, while being significantly suppressed by park perimeter‐area ratio and built‐up land patch proportion; abundance was core‐driven by flowering plant richness, patch quantity, and patch proximity; diversity relied on the synergistic effects of grassland patch proportion and plant diversity. This study confirms that under intensive urbanization, coordinated management of intra‐park habitat resources and surrounding landscape heterogeneity can significantly mitigate urban development‐induced butterfly diversity decline, providing a multi‐scale planning framework for establishing a “patch‐matrix” linkage ecological regulation system in high‐latitude cold‐region cities.

## Graphical abstract

Urban parks play a crucial role in biodiversity conservation amid increasing urbanization. This study investigates the effects of park characteristics and surrounding landscape heterogeneity on butterfly diversity in 44 urban parks in Harbin, China. Results show that integrating spatial heterogeneity metrics significantly improves model performance, providing key insights for urban biodiversity management and conservation.

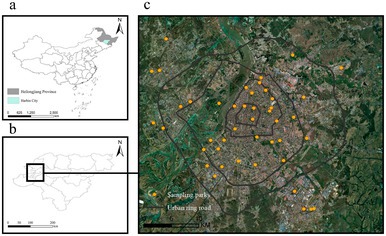

## 1 Introduction

Since the mid‐twentieth century, the rapid growth of the global population and the continuous expansion of human activities have brought unprecedented pressure on the ecological environment. *As a key driver of this pressure*, the global urbanization rate is expected to rise to 68% by 2050, with developed countries reaching 86% and China 71.2% (Citaristi 2022). Urbanization is reshaping biodiversity through multi‐scale mechanisms. At the global level, fragmentation of natural habitats and expansion of road networks are cutting continuous ecosystems into isolated patches, severely impeding species migration and gene flow. For example, satellite monitoring shows that tropical rainforests are being lost to urbanization at an average annual rate of 10 million hectares globally, exacerbating the islanding of endangered species (Hansen et al. 2013). Meanwhile, biomes are showing significant convergence: a few generalist species (e.g., the house sparrow *Passer domesticus* ) form dominant populations in cities around the globe, resulting in 70% of the individuals in Europe's urban bird communities consisting of only 15 species (Aronson et al. 2014). At the regional scale, imbalances in ecosystem functioning are particularly striking—the disappearance of top predators has triggered a proliferation of phytofauna populations, resulting in overconsumption of vegetation, while artificial light sources have disrupted insect circadian rhythms, leading to a 40% decline in the abundance of nocturnal pollinators in European cities (Knop et al. 2017). At the level of native adaptation, species have been forced to evolve unique survival strategies: the North American white‐crowned bunting ( *Zonotrichia leucophrys* ) penetrates urban noise by raising the dominant frequency of its calls (Brumm and Slabbekoorn 2005), and artificial microhabitats (e.g., rooftop gardens) reconfigure the gradient of biodiversity, with the species of urban bees in Berlin even outnumbering neighboring forests (Theodorou et al. 2020). Together, this evidence suggests that urban sprawl is threatening global biodiversity security.

Lepidoptera exhibit unique monitoring value in responding to urbanization stressors: their short generation cycles sensitively reflect environmental shifts, while larval‐stage host plant specificity and adult foraging mobility establish them as dual indicators of habitat connectivity and resource availability (Kuussaari et al. 2021). Research demonstrates that butterfly community composition changes along urban gradients explain 68% of landscape heterogeneity variation (Aronson et al. 2017). In the Tokyo Metropolitan Area, butterfly species richness strongly correlates with green patch aggregation within 500 m radii, their sensitivity qualifying them as early‐warning sentinels for urban habitat network health assessment (Jokimäki et al. 2020). China currently documents 2077 butterfly species, including 583 endemics, underscoring its status as a biodiversity hotspot (Mittermeier and Goettsch 1997; McNeely et al. 1990). However, rapid urban expansion induces unprecedented habitat fragmentation, jeopardizing urban‐adapted species. Recent national policies position biodiversity conservation as central to China's sustainable development strategy, with Lepidoptera prioritized as key arthropod conservation targets under the Ministry of Ecology and Environment's framework (Bergkamp and Xu 2017). Nevertheless, critical baseline data gaps persist in Northeast China's lepidopteran communities, severely impeding region‐specific conservation planning.

Urban parks help mitigate the negative impacts of rapid urbanization on insects and birds (Aronson et al. 2014). According to the literature from the Cerrado region of Brazil (Hayes et al. 2023), urban parks can protect a wide range of species, including insects and birds, with a number of species that exceeds the diversity of the surrounding natural areas. A study of urban parks in Olomouc, Czech Republic, found that although these parks are man‐made habitats, they play a crucial role in maintaining bird diversity. The findings also emphasized the importance of certain native vegetation structures (e.g., ancient trees and shrub ecotones) in urban parks for the conservation of urban bird biodiversity (Machar et al. 2022; Leveau et al. 2022). Similarly, 74 species of butterflies were recorded in the urban parks of the Kozhikode community in India, which exceeded the number of species in the surrounding natural areas (Leveau et al. 2022). A study of East Jakarta Administrative City and Pontianak City showed that urban parks play a key role in butterfly conservation as green open spaces (Gonzaga 2023). These urban parks not only provide essential nectar plants and sheltering vegetation for butterflies, but also support butterfly habitats through their clustered configuration (Azahra et al. 2022). Urban parks serve as biodiversity refuges with great conservation potential for a wide range of taxa (Norton et al. 2016; Sattler et al. 2010). Urban greening managers and city planners need to recognize this potential (Alvey 2006).

Furthermore, exploring the main factors influencing the structure and composition of butterfly species communities is essential for developing biodiversity conservation, monitoring, and management strategies (Kumar et al. 2009). Current research suggests that butterfly species diversity may be influenced by local features or landscape characteristics (Ombugadu et al. 2023; Lin et al. 2023; Horak et al. 2022). For example, a study conducted in Rocky Mountain National Park, USA, found strong positive correlations between butterfly species richness and plant species richness, proportion of shrubland, and mean patch size, suggesting that butterfly richness is more closely related to local features within the study area (Kumar et al. 2009). Similarly, a report from the Kihansi Gorge, Tanzania, highlighted a strong positive correlation between habitat characteristics and butterfly species and community structure. The researchers suggested that maintaining habitat quality should be a priority for the conservation of butterfly communities in the region. On the other hand, some studies have shown that urban butterfly diversity is more influenced by surrounding landscape features. Pendl et al. (2022) explored the relationship between butterfly species composition and local and landscape features in private gardens in Vienna, Austria. They found that the number of butterfly species was positively correlated with the type of land in the surrounding landscape that favored butterflies but negatively correlated with the proportion of woodland. In central France, a study using generalized linear mixed models to relate butterfly species richness to landscape variables within a radius of 250–5000 m found that butterfly communities were influenced by landscape composition, structure and connectivity (Archaux et al. 2018). However, further research is needed to examine whether butterflies in parks are influenced by both the local characteristics of the park and the characteristics of the landscape.

Understanding how spatial heterogeneity affects ecological patterns and processes is one of the main focuses of landscape ecology. Therefore, it is important to introduce the concept of spatial heterogeneity in order to gain insights into how landscape features in the urban periphery affect butterflies in parks. A study by Yuguo Qian and colleagues showed that incorporating spatial heterogeneity helps to understand the various landscape elements around the city and helps to plan butterfly parks to effectively meet habitat requirements (Qian et al. 2020). However, spatial heterogeneity is a complex and highly scale‐dependent phenomenon that is difficult for ecologists to accurately quantify at different scales (Kolasa and Rollo 1991; Fortin and Agrawal 2005; Wagner and Fortin 2005; Gustafson 1998). This complexity stems from the interactions between various biotic and abiotic factors in ecosystems and the different responses of organisms to these factors (Milne 1991; Huston 1994). Different organisms respond to spatial heterogeneity at multiple scales, depending on their scale of perception (Levins 1968), the spatial structure of the landscape (Godron 1986), and their natural life history. Therefore, identifying the dominant scales at which species respond to these factors is crucial for biodiversity conservation and management.

Current research on the impacts of urban landscape spatial heterogeneity on butterflies in parks remains relatively limited. Kumar (Norton et al. 2016), Davis (Huston 1994), Bergman (Levins 1968), and colleagues attempted to determine the dominant scale of butterfly responses to spatial heterogeneity by comparing *R* ^2^ values across different spatial extents. Kumar et al. (2009) investigated the relationship between butterfly richness and spatial heterogeneity using multiple linear regression. Multiple linear models, known for their computational simplicity, have been widely adopted across scientific disciplines due to their strong versatility (Jiang 2022). However, these models assume that data or residuals follow a normal distribution, a premise that does not hold for non‐normally distributed data such as butterfly richness (Vishwakarma et al. 2023). Additionally, as the number of independent variables increases, multiple linear models are prone to overfitting, which reduces model performance (Fleishman et al. 2002). Although a few studies have quantified the effects of multi‐scale spatial heterogeneity on butterfly communities, they failed to fully integrate landscape spatial heterogeneity factors (Fleishman et al. 2002; Collinge et al. 2003; Krauss et al. 2003; Strathmann 2005). Existing literature predominantly focuses on single dimensions: for example, Han et al. (2021) only incorporated the proportion of green vegetation within 150–3000 m radii (a landscape composition metric) into generalized linear models. In contrast, this study comprehensively analyzes urban landscape spatial heterogeneity by considering both landscape composition and configuration of surrounding areas, thereby more accurately identifying the mechanisms of landscape heterogeneity driving butterfly diversity changes. By employing AICc‐selected optimal models, this research determines the dominant scale of butterfly responses. Compared to methods relying solely on *R* ^2^ values, this approach helps avoid overfitting and enhances model predictive power, thus more precisely reflecting the impacts of urban park landscape characteristics on butterflies (Kamalov et al. 2023). Furthermore, the adoption of generalized linear mixed‐effects models (GLMMs)—through specifying response variable distribution families and incorporating random effects—simultaneously addresses non‐normal data and non‐independent observations, demonstrating greater ecological applicability than traditional linear models (Zuur et al. 2009).

This study examines the impact of urbanization on butterfly diversity in the urban area of Harbin, northern China. By incorporating a spatial heterogeneity index into the model, we aimed to improve and enhance the traditional understanding and prediction of butterfly diversity, which often relies solely on park variables or various land cover areas. The study hypothesized that spatial heterogeneity in urban landscapes affects butterfly communities and that the inclusion of a spatial heterogeneity index would improve the predictive power of the model. The study had three objectives: (1) to investigate how butterflies respond to spatial heterogeneity at different scales; (2) to identify the spatial scales in urban areas where butterflies responded most strongly to the heterogeneity index; and (3) to validate whether the spatial heterogeneity index improves the performance of the model.

## 2 Materials and Methods

### 2.1 Study Area and Sampling Sites

**FIGURE 1:**
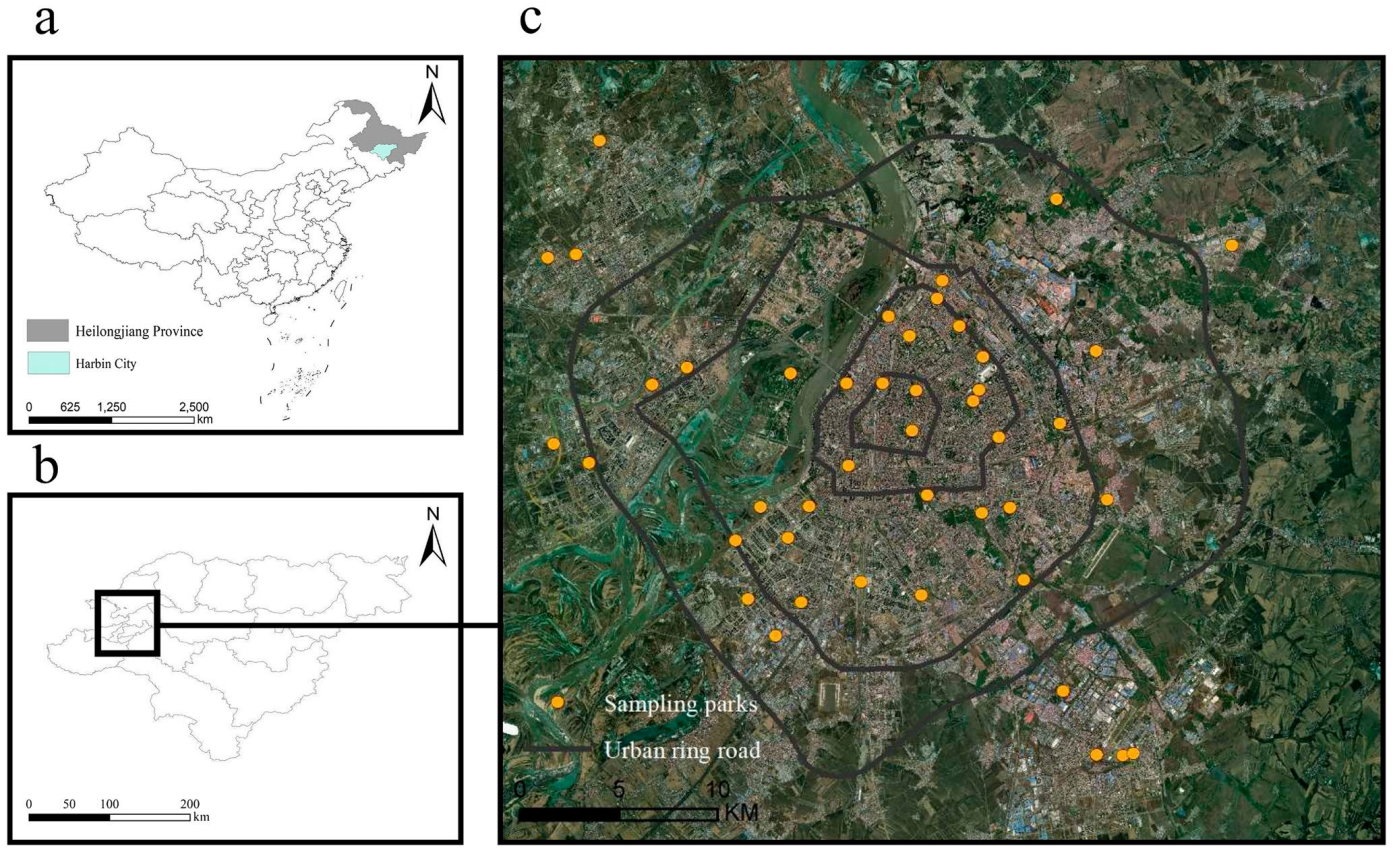
Location map of sampling parks in Harbin, China. (a) Administrative boundaries of China (including national borders); (b) Administrative boundaries of Heilongjiang Province with the location of Harbin City; (c) Distribution of sampling parks and urban ring roads in Harbin's main urban area.

The study area is the urban region of Harbin, located in the southern part of Heilongjiang Province, China, between 125°42′ and 130°10′ E longitude and 44°04′ and 46°40′ N latitude. The city spans an urban area of 10,198 km^2^, characterized by flat terrain with an average elevation of 151 m. Harbin experiences a mid‐temperate continental monsoon climate, with long winters and short summers. The average annual precipitation is 569.1 mm, with 60% of the total precipitation in summer between June and September. Snowfall is concentrated from November to January. Harbin has distinct seasons, with an average temperature ofaround–199°C in January and approximately 23°C in July. Harbin's urban built‐up area contains numerous parks, while the surrounding regions are rich in farmland and woodland, providing diverse plant species that offer abundant food and habitat for butterflies. We conducted field surveys in 44 urban parks, evenly selected in a radial pattern from within the first ring road to outside the fourth ring road. Park size ranges from 1.47 to 210.94 ha, with 3–14 parks within each ring, as shown in Figure 1.

### 2.2 Butterfly Sampling

Before the formal survey, a preliminary field investigation was conducted to classify all green spaces in the parks into microhabitats, with each designated area serving as a sampling point. Based on vegetation structure and management practices, a total of 13 microhabitat types were identified through field surveys across all the parks: (1) Trees: habitats covered solely by trees; (2) Trees + Shrubs: habitats with both trees and shrubs; (3) Trees + Grasses: habitats with both trees and grass cover; (4) Trees + Shrubs + Grasses: habitats with trees, shrubs, and grass cover; (5) Shrubs: habitats covered solely by shrubs; (6) Shrubs + Grasses: habitats with both shrubs and grass cover; (7) Grasses: habitats covered solely by grass; (8) Unmanaged Area + Trees: unmanaged habitats that include trees; (9) Unmanaged Area + Trees + Shrubs: unmanaged habitats with trees and shrubs; (10) Unmanaged Area + Trees + Grasses: unmanaged habitats with trees and grass; (11) Unmanaged Area + Trees + Shrubs + Grasses: unmanaged habitats with a combination of trees, shrubs, and grasses; (12) Unmanaged Area + Shrubs + Grasses: unmanaged habitats with shrubs and grasses; and (13) Unmanaged Area + Grasses: unmanaged habitats covered solely by grass.

Based on preliminary habitat classification results, sampling points were established near the central area of each habitat type. Using the time‐constrained sampling method (Kadlec et al. 2012; Dallimer et al. 2012), each sampling point underwent 15 min of active searching within a 20‐m radius. Butterfly surveys were conducted during flight seasons (June to September) in 2022 and 2023, with two monthly replicates per sampling site. Replicates were separated by strictly ≥ 10‐day intervals to avoid pseudoreplication, exceeding the empirically derived dispersal thresholds of dominant butterfly species (e.g., Pieridae such as *Pieris rapae* exhibit an average dispersal distance of 1.2 km, with a maximum range of up to 1.5 km within 10 days under extreme conditions; Stevens et al. 2010), thereby ensuring complete vacating of individuals from the sampling area. Butterfly activity is influenced by solar radiation, wind speed, and cloud cover. Increased flight activity occurs with higher temperatures, stronger sunlight, and reduced cloudiness (Evans et al. 2019). Surveys were thus restricted to clear or near‐clear days with temperatures above 15°C between 08:30 and 17:00. To ensure effective coverage of butterfly communities, the survey sequence across parks was alternated between monthly replicates, aiming to sample each park at different times of day. This approach balances practicality and efficiency under personnel and time constraints. For example, in the first survey of each month, parks A and B were surveyed in the morning, while parks C and D were surveyed in the afternoon. In the second survey of the month, parks C and D were scheduled for the morning, while parks A and B were surveyed in the afternoon.

During the initial identification phase, butterflies were classified based on macroscopic morphological characteristics such as wing venation patterns and flight posture, targeting only unambiguous common species in the Chinese Lepidoptera checklist, followed by immediate release. Specimens that remained unidentified during this rapid assessment were captured, preserved in numbered triangular envelopes, and transported to the laboratory for specimen preparation. The identification process relied on “Monograph of Chinese Butterflies” (Volumes 1 and 2), Illustrated Book of “Butterflies of China” (all four volumes), and the National Animal Specimen Resource Center (http://museum.ioz.ac.cn/index.html).

### 2.3 Environmental Variables Data Collection

This study selected a total of 41 environmental variables, reflecting the park scale (7 variables) and landscape scale (34 variables) of the parks.

Park variables: Seven park variables were selected based on park attributes and resources: (1) area of the park (ha) (Area): the area of the surveyed region within the park; (2) perimeter‐area ratio of the park (P.A): the ratio of the park's perimeter to its area, reflecting the complexity of the park's shape; (3) age of the park (Age): defined as the time from the establishment of the park to 2022; (4) distance from city center (Dis): the straight‐line distance from the park's center to the city center; (5) flowering plant richness (Flo): the total number of flowering plant species in the shrub and herb layers at each sample point within the park, indicating the availability of potential nectar plants; (6) overall plant species richness (Plant): the total number of plant species in the tree, shrub, and herb layers at each sample point within the park, indicating the availability of potential host plants and adult butterfly food sources; and (7) Shannon index of park plants (PlantS): Shannon diversity index calculated from the number of individuals and species in the tree, shrub, and herb layers at each sample point within the park, using Paleontological Statistics (PAST) software (Hammer and Harper 2001). Descriptive statistics for these variables across all parks are summarized in Table S1.

Monthly surveys documented flowering plant species richness at each sampling site. Total plant species richness was surveyed during the peak growing season (July–August) using a hierarchical nested sampling protocol: within a 20 × 20 m primary quadrat, arboreal‐layer vegetation (woody plants exceeding 4 m height) was inventoried across the entire plot; shrub‐layer vegetation (woody plants ≤ 4 m height) was recorded through four uniformly distributed 5 × 5 m subplots; herbaceous‐layer vegetation (herbaceous plants ≤ 1.5 m height) was assessed via five evenly spaced 1 × 1 m subplots. Total species richness was calculated as the cumulative count of unique species across all three strata.

Landscape variables: To describe the impact of landscape characteristics surrounding parks on butterfly richness, we quantified the landscape composition and configuration at different spatial scales around the parks. Selecting the appropriate scale for quantifying spatial heterogeneity is a challenging task, as spatial heterogeneity is highly scale‐dependent. Most ecologists quantify spatial heterogeneity based on the activity range of species. However, for community‐level studies, it is difficult to determine the scale at which the community or species shows the strongest response due to variations in species' dispersal abilities, life histories, and habitat requirements (Kumar et al. 2009). Based on related research on butterfly dispersal (Wahlberg et al. 2002; Schneider 2003), we used ArcGIS 10.8 software to create buffers with radii of 100, 200, 350, 500, 750, 1000, 1500, and 2500 m around each park, which served as the spatial scales for further quantifying spatial heterogeneity. Land use data for the study area was obtained from the European Space Agency (ESA) WorldCover project's 2021 WorldCover map (esa‐worldcover.org), with a resolution of 12 m, used to define different patch types. The projection was WGS_1984_UTM_Zone_45N. Land use data provided the essential input layer for the calculation of landscape metrics. Using this data, we quantified 34 landscape pattern indices at different spatial scales around the parks. These indices included the percentage of landscape of woods, buildings, meadows, crops, waterbodies, and bare ground (PLAND), the mean patch area of woods, buildings, meadows, crops, waterbodies, and bare ground (A_MN), the area‐weighted mean patch area of woods, buildings, meadows, crops, waterbodies, and bare ground (A_AM), number of patches (NP), patch density (PD), largest patch index (LPI), edge density (ED), mean patch area (AREA_MN), area‐weighted mean patch area (AREA_AM), mean shape index (SHAPE_MN), mean fractal dimension index (FRAC_MN), mean Euclidean nearest neighbor distance (ENN_MN), mean edge contrast index (ECON_MN), interspersion juxtaposition index (IJI), patch cohesion index (COHESION), patch richness (PR), patch richness density (PRD), Shannon's diversity (SHDI), and Simpson's diversity (SIDI). These indices were referenced from the study by Kumar et al. (2009). They represent five fundamental components of spatial heterogeneity (SH): (1) the number of patch types (PR and PRD); (2) the proportion of each patch type (PLAND); (3) the spatial arrangement of patches (NP, PD, IJI, COHESION, and ENN_MN); (4) patch shapes (SHAPE_MN and FRAC_MN); and (5) the contrast between adjacent patches (LPI, ED, ECON_MN, AREA_MN, AREA_AM, A_MN, and A_AM). The selection of indices was based on their potential biological relevance to butterfly species richness (Collinge et al. 2003; Davis et al. 2007; Mazerolle and Villard 1999; Kumar et al. 2006).

### 2.4 Defining Butterfly Community Metrics as Response Variables: Species Richness, Abundance, and Shannon Diversity

To investigate the effects of park characteristics and landscape patterns on butterflies, we selected butterfly species richness, abundance, and Shannon diversity index as response variables, as these metrics effectively reflect community structure and composition. These indices serve as valuable ecological indicators, enhancing research reliability and scientific rigor through field observations and comparisons with existing studies (Lin et al. 2023). They effectively capture the impacts of park features and landscape configurations on butterfly biodiversity, ensuring interpretable and generalizable findings (Guariento et al. 2023) Furthermore, their suitability for multi‐scale spatial analysis enables assessment of landscape heterogeneity effects across varying spatial extents, providing critical insights into urban ecosystem dynamics (Lin et al. 2023). By employing species richness, abundance, and Shannon diversity as core metrics, we quantitatively evaluate environmental influences on butterfly communities, establishing clear and measurable benchmarks for ecological analysis.

### 2.5 Statistical Analysis

This study uses Welch's ANOVA to analyze the differences in richness across different urban areas, followed by Games‐Howell post hoc comparisons. Spatial patterns of butterfly community composition along the urban gradient were further analyzed using Non‐metric Multidimensional Scaling (NMDS).

Before analysis, we performed the Shapiro–Wilk test for normality on all variables. Due to some data not conforming to a normal distribution even after transformation, we used Spearman's correlation coefficient (r) to investigate the relationship between butterfly richness and heterogeneity indices across eight spatial scales. Following variable screening, we initially eliminated multicollinearity by examining the correlations among all predictor variables, retaining only one variable from groups of variables with correlations greater than 0.7, selecting the variable with the highest correlation with butterfly richness. Further multicollinearity diagnosis was performed using the Variance Inflation Factor (VIF), considering VIF < 5 as an indication of no multicollinearity issues.

This study was conducted using R version 4.4.1. Generalized linear mixed models (GLMMs) were constructed via the glmmTMB package, with negative binomial and Gamma distributions applied to analyze responses of butterfly richness, abundance, and diversity to predictors. Park ID and month were included as random effects to account for non‐independent observations. All potential variable combinations were screened through full‐subset regression using the MuMIn package, with optimal model selection based on the small‐sample corrected Akaike Information Criterion (AICc) (Burnham 1998). Building on this framework, although computationally intensive, full‐subset regression ensures identification of the most accurate explanatory models. AICc adjusts for sample size effects on model selection, improving robustness in small‐sample scenarios. By balancing model fit and complexity, AICc enhances interpretability and predictive performance. Models with ΔAICc < 2 were retained as the best‐fitting model set (Si et al. 2014), where ΔAICc represents the difference between a model's AICc value and that of the top‐ranked model (i.e., the model with the minimum AICc). Finally, model explanatory power was quantified using marginal R^2^ (R^2^m) and conditional R^2^ (R^2^c) computed via the performance package.

## 3 Results

### 3.1 Butterfly Species Composition and Distribution

During a two‐year survey of butterfly diversity in 44 urban parks, we recorded a total of 12,885 butterflies, encompassing 5 families and 38 species. The number of species recorded in each park varied, with species richness ranging from 1 to 22 species. The most abundant species was *Pieris rapae* (Pieridae), accounting for 29% of the total, 3760 individuals, followed by *Everes argiades* (Lycaenidae), with 1845 individuals, 14%. Other notable species included *Polygonia c‐aureum* (Nymphalidae), 1441 individuals, and *Colias erate* (Pieridae), 1695 individuals. The individual counts for all other species did not exceed 1000 (see Appendix S1). *Pieris rapae* was recorded in 43 parks. *Everes argiades* and *Polygonia c‐aureum* were also widely distributed, observed in 34 and 38 parks, respectively. Ten species were recorded in only a single park.

In Harbin, the study identified five distinct urban areas: within the First Ring Road, Second Ring Road, Third Ring Road, Fourth Ring Road, and areas beyond the Fourth Ring Road. Within the First Ring Road, three parks recorded 6 butterfly species, 22 individuals. In the Second Ring Road, 10 species were observed across eight parks, 364 individuals. In the Third Ring Road, 36 species were documented in 14 parks, 7410 individuals. In the Fourth Ring Road, 27 species were recorded in eight parks, 2415 individuals. Beyond the Fourth Ring Road, 23 species were documented in 10 parks, 2482 individuals. The species *Polygonia c‐album*, *Pieris rapae* , *Polygonia c‐aureum*, and *Everes argiades* were recorded across all urban areas (see Table S2).

**FIGURE 2:**
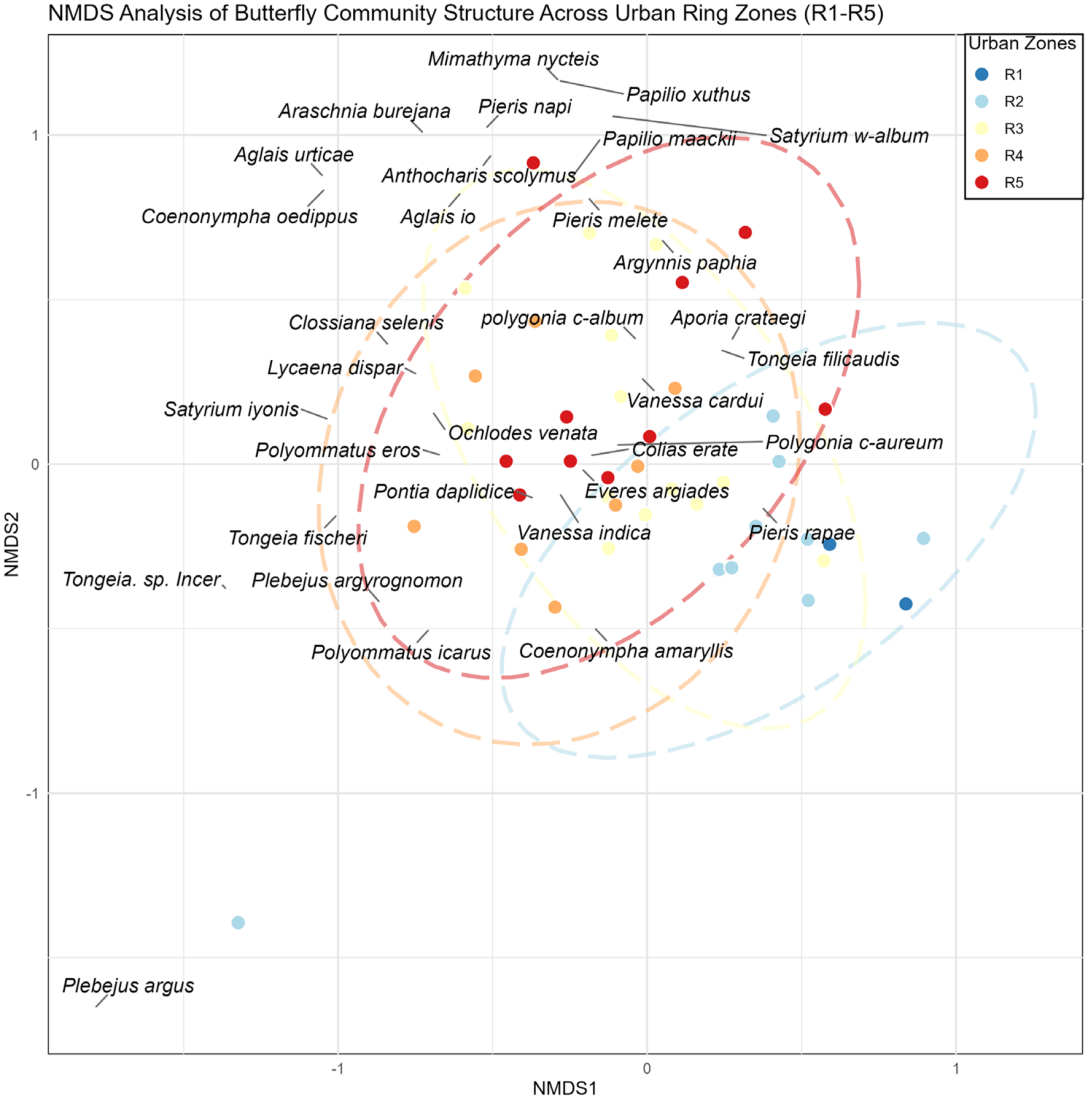
NMDS analysis of butterfly community structure across urban ring zones (R1–R5). R1 is parks inside the first ring, R2 is parks inside the second ring, R3 is parks inside the third ring, R4 is parks inside the fourth ring, and R5 is parks outside the fourth ring.

**FIGURE 3:**
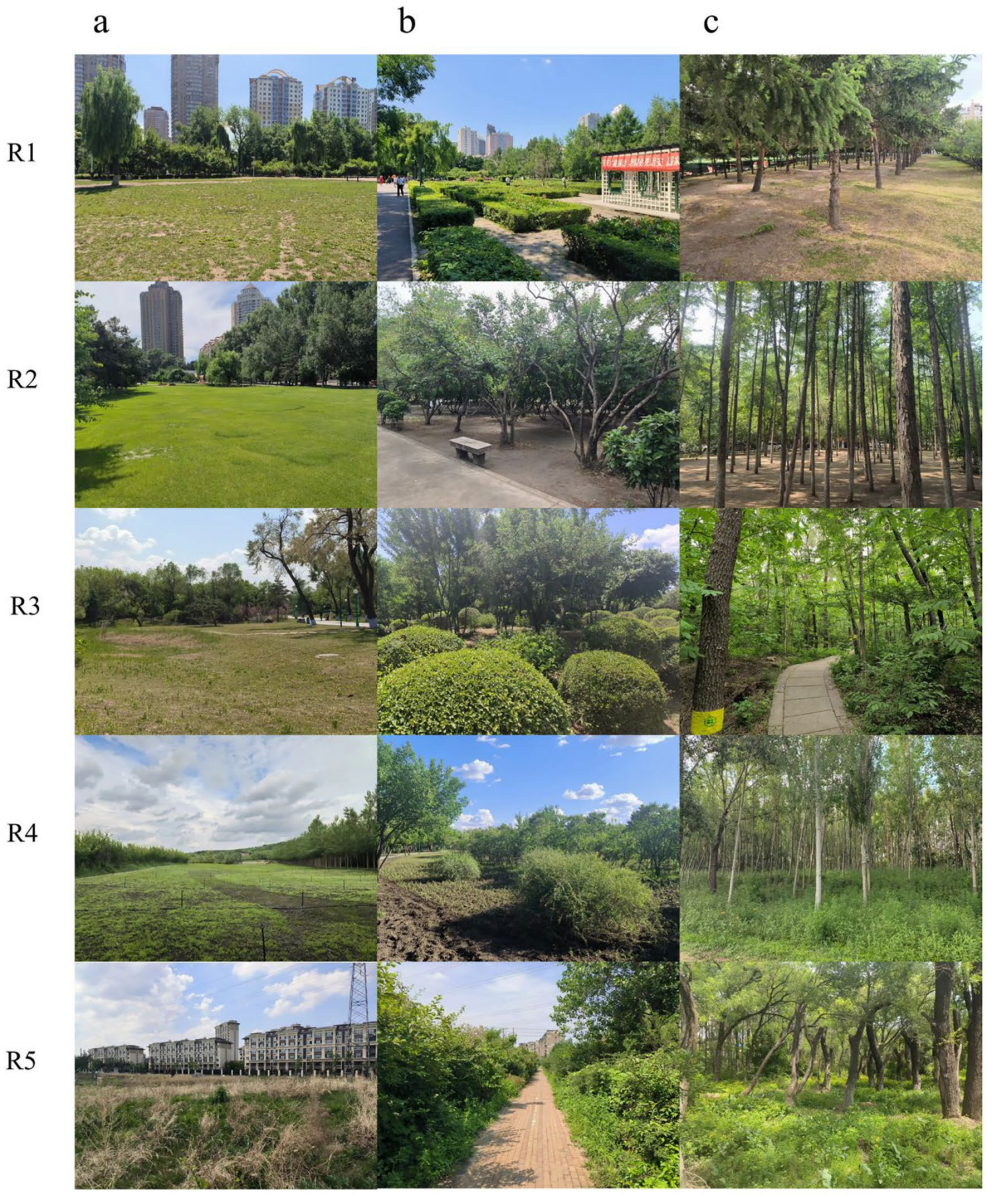
Comparison of urbanization levels across parks. (a) Grassland habitat, (b) Shrubland habitat, (c) Woodland habitat. R1 is parks inside the first ring, R2 is parks inside the second ring, R3 is parks inside the third ring, R4 is parks inside the fourth ring, and R5 is parks outside the fourth ring.

The NMDS ordination (Figure 2) revealed distinct spatial clustering: parks within the First and Second Ring Roads (R1, R2) were concentrated in the central‐right portion of the ordination space, largely separated from species points. Only the generalist *Pieris rapae* overlapped spatially with these inner‐city parks. In contrast, most species points clustered near and around parks in the outer rings (R3–R5), indicating stronger associations between butterfly diversity and peri‐urban green spaces. This divergence in species‐habitat associations likely stems from urbanization‐driven habitat modifications: inner‐ring parks (R1–R2) exhibited intensive management practices and degraded vegetation conditions, whereas outer‐ring parks (R3–R5) retained more heterogeneous and self‐sustaining habitats (Figure 3).

**FIGURE 4:**
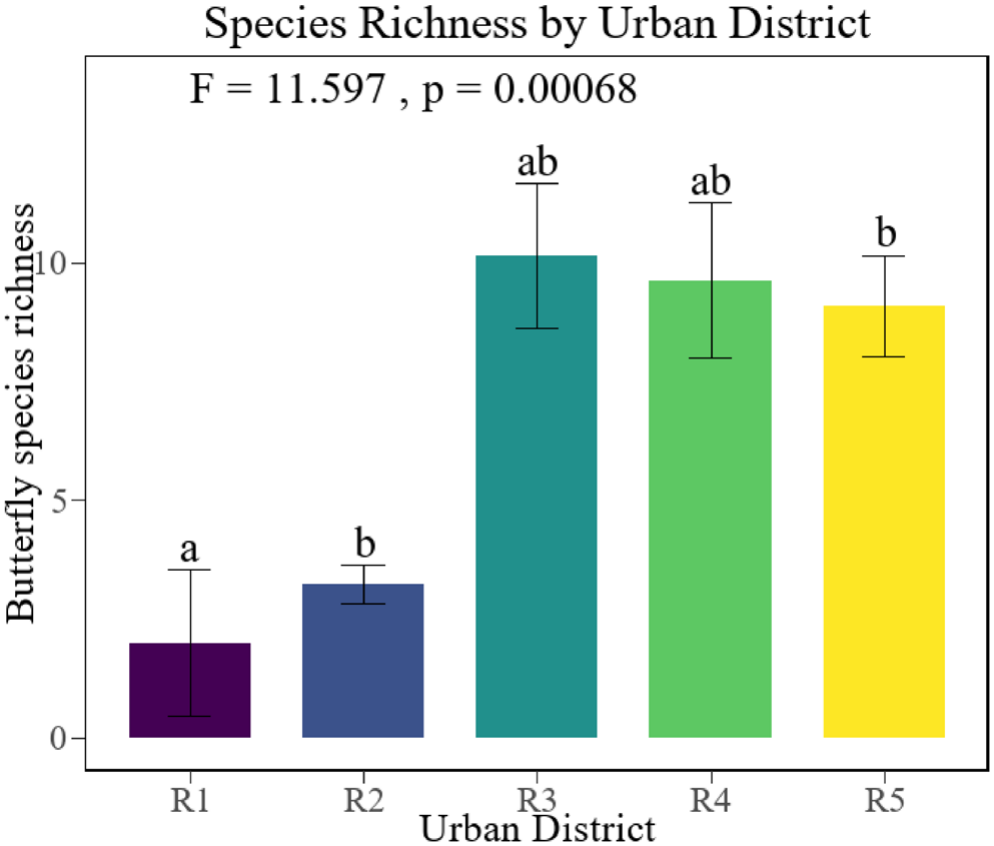
Butterfly species richness in urban areas. The letters (a, b, ab) in the figure indicate significant differences between groups: Groups with the same letter (e.g., a and ab) are not significantly different from each other, while groups with different letters (e.g., a and b) are significantly different. The whisker lines represent data variability, usually indicating the standard error (SE). R1 is parks inside the first ring, R2 is parks inside the second ring, R3 is parks inside the third ring, R4 is parks inside the fourth ring, and R5 is parks outside the fourth ring.

Welch ANOVA analysis results indicated significant differences in butterfly richness across different urban areas (*p* < 0.001), as depicted in Figure 4. The Games‐Howell test results revealed significant differences in butterfly richness between the second ring and the third ring (*p* = 0.0044), the second ring and the fourth ring (*p* = 0.0322), and the second ring and outside the fourth ring (*p* = 0.0019). Furthermore, a significant difference in butterfly richness was observed between the first ring and the third ring (*p* = 0.0401). These findings suggest that as the distance of parks from the city center increases, the overall number of butterfly species tends to increase. This trend is likely due to the presence of more green patches in peripheral areas, which provide habitats and migration pathways for butterflies.

### 3.2 The Relationship Between Butterflies and Various Environmental Factors

**FIGURE 5:**
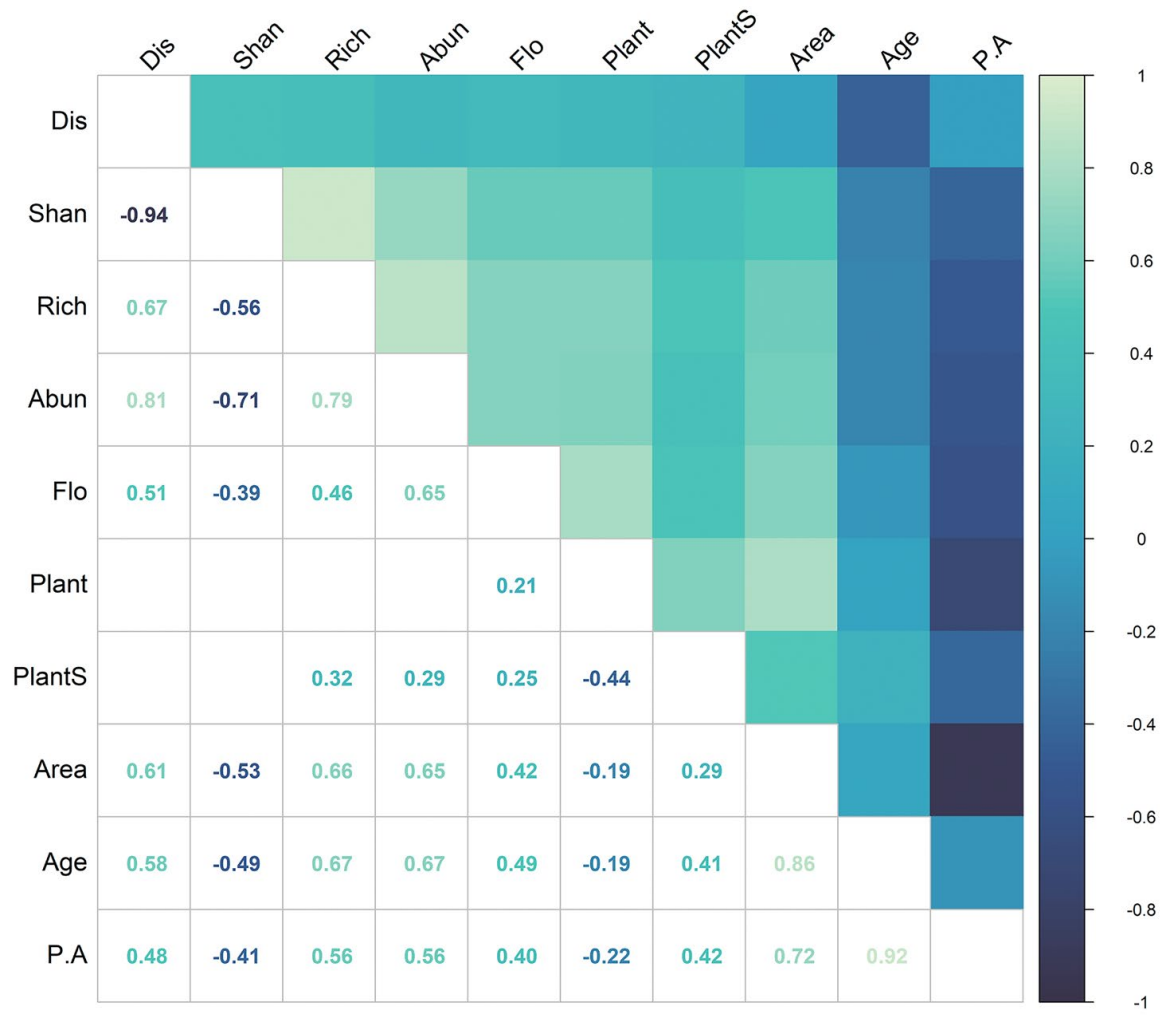
Heatmap of correlations between butterfly diversity metrics and park attributes.***p < 0.001; **p < 0.01; *p < 0.05. Rich: Butterfly species richness; Abun: Butterfly abundance; Shan: Butterfly Shannon diversity index; Area: Park area (ha); PA: Park perimeter‐area ratio; Flo: Abundance of blooming plants; Plant: Total plant abundance; PlantS: Plant Shannon diversity index; Age: Park age (years); Dis: Distance to city center (km).

Spearman's correlation analysis revealed relationships between butterfly community metrics (abundance, richness, and Shannon diversity) and park characteristics (Figure 5). Due to multicollinearity among variables, specific indicators were excluded from the final models.

Flowering plant richness (Flo) exhibited strong correlations with butterfly abundance (*r* = 0.66, *p* < 0.01) and species richness (*r* = 0.67, *p* < 0.01). Park area showed significant positive associations with butterfly abundance (*r* = 0.61, *p* < 0.01), species richness (*r* = 0.58, *p* < 0.01), and Shannon diversity (*r* = 0.48, *p* < 0.01), indicating that larger parks support more abundant and diverse butterfly communities. Plant Shannon diversity (PlantS) moderately correlated with butterfly richness (*r* = 0.49, *p* < 0.01) and diversity (*r* = 0.40, *p* < 0.01), suggesting that floral niche differentiation may drive community assembly. Distance from the city center (Dis) weakly correlated with richness (*r* = 0.41, *p* < 0.01) and diversity (*r* = 0.42, *p* < 0.01), though ecological interpretability remained limited. Park age demonstrated no significant associations with any metrics (∣*r*∣ < 0.22, *p* > 0.05).

The impacts of land cover composition on butterfly community structure and composition exhibited complex scale‐dependent patterns. Built‐up land coverage (PLAND_Built) showed strong inhibitory effects on butterfly abundance, richness, and diversity, with the strongest suppression on richness at the 100 m scale (*r* = −0.611), persisting at 2400 m (*r* = −0.526), indicative of long‐term habitat loss from urbanization. PLAND_Built negatively correlated with abundance (*r* = −0.511 at 100 m to *r* = −0.416 at 2400 m, *p* < 0.001) and diversity (weakening with scale), reflecting localized habitat homogenization. Mean built‐up patch area (A_MN_Built) strongly reduced abundance (*r* = −0.568) and richness (*r* = −0.474) at 200 m, highlighting ecological isolation by large impervious patches. Natural/semi‐natural habitats exhibited type‐specific roles: Forest coverage (PLAND_Tree) strongly supported richness (*r* = 0.558, *p* < 0.001 at 500 m) and consistently enhanced abundance (*r* = 0.392–0.477) and diversity (*r* = 0.428–0.500). Cropland coverage (PLAND_Crop) maximally drove richness at 350 m (*r* = 0.515), while its patch size (A_MN_Crop) improved diversity at 2400 m (*r* = 0.498), suggesting agricultural coherence facilitates niche differentiation. Water coverage (PLAND_Water) locally boosted abundance at 100 m (*r* = 0.477) but lacked broad‐scale effects due to fragmentation. Grassland coverage (PLAND_Grass) moderately influenced diversity (*r* = 0.345–0.419), with patch size (A_MN_Grass) enhancing abundance at 200 m (*r* = 0.369), underscoring grasslands' transitional habitat value (see Figure S1).

Landscape spatial configuration metrics triggered nonlinear and scale‐specific responses. Patch number (NP) maximally enhanced richness at 350 m (*r* = 0.702), indicating moderate fragmentation promotes species coexistence through edge interfaces. Edge density (ED) positively correlated with richness (*r* = 0.438, *p* < 0.001 at 350 m) and abundance (peak *r* = 0.470 at 350 m, declining to *r* = 0.28 at 2400 m, *p* < 0.001), but weakly with diversity (*r* = 0.362 at 100 m, *p* < 0.001). Largest patch index (LPI) strongly suppressed richness (*r* = −0.675 at 350 m, *p* < 0.001), abundance (*r* = −0.404 at 100 m, *p* < 0.001), and diversity (*r* = −0.630 at 350 m, *p* < 0.001). Shannon landscape diversity (SHDI) positively influenced richness (*r* = 0.527 at 100 m to *r* = 0.496 at 2400 m) and diversity (*r* = 0.490–0.485), reflecting cumulative habitat heterogeneity, while moderately associating with abundance (*r* = 0.449–0.392). Fractal dimension (FRAC_MN) strengthened richness (*r* = 0.442 at 350 m to *r* = 0.474 at 2400 m, *p* < 0.001) and diversity (*r* = 0.343–0.398), demonstrating complex patch shapes sustain biodiversity across scales (see Figure S2).

### 3.3 Optimal Generalized Linear Model for Butterfly Richness

Model selection based on the corrected Akaike Information Criterion (AICc) identified optimal spatial scales for butterfly species richness (100 m), abundance (200 m), and Shannon diversity (200 m) (Table 1; see Table S3 for multi‐scale model comparisons). Conditional *R* ^2^ values varied significantly, with abundance models showing the highest explanatory power (R^2^c = 0.811), while diversity models exhibited minimal unobserved spatial heterogeneity (park SD ≈ 0).

**TABLE 1:** Generalized linear mixed model (GLMM) parameter estimation for butterfly community indicators based on AICc screening”.

Butterfly metric	Range	Fixed effects	Estimate	Std. error	*z* value	Pr(>|z|)	Random effects (variance ± SD)
Richness	100 m	(Intercept)	1.162	0.088	13.174	< 2e‐16	Park: 0.064 ± 0.253
		P.A	−0.337	0.084	−3.996	6.44e‐05	Month: 0.016 ± 0.126
		Flo	0.130	0.060	2.187	0.0288	
		Dis	0.140	0.065	2.161	0.0307	
		PLAND_Built	−0.295	0.006	−4.923	2.55e‐05	
Abundance	200 m	(Intercept)	3.28	0.302	10.848	< 2e‐16	Park: 0.518 ± 0.720
		Flo	0.527	0.118	4.478	7.55e‐06	Month: 0.301 ± 0.549
		Dis	0.445	0.132	3.381	0.000721	
		NP	0.635	0.134	4.736	2.18e‐06	
		ENN_MN	0.456	0.134	3.402	0.000669	
Diversity	200 m	(Intercept)	−0.385	0.097	−3.985	6.75e‐05	Park: ≈0
		Plant	0.476	0.097	4.900	9.57e‐07	Month: 0.015 ± 0.122
		Dis	0.376	0.104	3.619	0.000977	
		PLAND_Grass	0.353	0.107	3.297	0.036330	
		A_MN_Built	0.265	0.127	2.093	0.070727	
		PR	0.338	0.113	2.988	0.002812	

The optimal model for butterfly species richness was identified at the 100 m scale (AICc = 696.5), with a marginal R^2^ of 0.572 and conditional *R* ^2^ of 0.678. Standardized regression coefficients revealed significant predictors: perimeter‐area ratio (P.A, β = −0.337, *p* < 0.001), flowering plant richness (Flo, β = 0.130, *p* = 0.029), distance to city center (Dis, β = 0.140, *p* = 0.031), and built‐up land coverage (PLAND_Built100, β = −0.295, *p* < 0.001). Each standard deviation increase in built‐up land coverage reduced richness by 29.5%, indicating micro‐scale habitat compression from urbanization. Flo promoted species coexistence through resource provisioning, while the positive effect of Dis suggested reduced anthropogenic pressures (e.g., traffic pollution, heat island effects) in peri‐urban parks. Random effects analysis showed spatial heterogeneity between parks (SD = 0.253) exerted 2.01 times stronger influence on richness than monthly fluctuations (SD = 0.126), highlighting spatial heterogeneity's dominance.

The optimal model for butterfly abundance at the 200 m scale (AICc = 1590.1) explained 81.1% of variation (conditional R^2^ = 0.811). Key predictors included flowering plant richness (Flo, β = 0.527, *p* < 0.001), number of patches (NP_200, β = 0.635, *p* < 0.001), and mean Euclidean nearest‐neighbor distance (ENN_MN_200, β = 0.456, *p* < 0.001). A one standard deviation increase in NP_200 increased abundance by 63.5%, confirming resource complementarity in moderately fragmented landscapes. ENN_MN_200's positive effect implied cross‐patch foraging by dominant species. Unobserved spatial heterogeneity contributed 54.3% of variance explanation (park SD = 0.720 vs. month SD = 0.549), suggesting localized management could regulate population size.

The optimal model for Shannon diversity at the 200 m scale (AICc = 238.5) showed marginal R^2^ = 0.379 and conditional R^2^ = 0.388. Significant predictors were plant species richness (Plant, β = 0.476, *p* < 0.001), mean grassland patch area (AREA_MN_Grass200, β = 0.353, *p* = 0.002), built‐up mean patch area (AREA_MN_Built200, β = 0.265, *p* = 0.036), bare land area‐weighted mean patch area (AREA_AM_Bare200), and patch richness (PR_200, β = 0.338, *p* = 0.003). AREA_MN_Grass200 and Plant promoted niche differentiation with standardized effects of 35.3% and 47.6%, respectively. PR_200 increased diversity by 33.8% per standard deviation, validating landscape heterogeneity's positive role. The counterintuitive weak positive effect of AREA_MN_Built200 may relate to ornamental flora filtering disturbance‐tolerant species rather than the ecological benefits of built‐up areas. Temporal fluctuations dominated random effects (month SD = 0.122; park SD ≈ 0).

## 4 Discussion

### 4.1 Spatial Distribution of Butterfly Species Richness

This study revealed a significant ring‐road gradient in butterfly diversity across Harbin's urban parks: third‐ring parks, hosting 36 species (94.7% of total species richness), emerged as biodiversity hotspots, significantly surpassing fourth‐ring (27 species) and outer fourth‐ring areas (23 species). This pattern likely stems from the synergistic effects of higher park density (14 parks) and superior habitat quality (low‐intensity management, high plant diversity) within the third ring. Overall, parks in urban fringe areas (third‐ring, fourth‐ring, and outer fourth‐ring) exhibited significantly higher species richness and abundance compared to central urban areas (first‐ and second‐ring), supporting the hypothesis that “butterfly richness is higher in urban fringe zones than in city centers.” While the initial hypothesis of “increasing richness with decreasing urbanization” held true from the first to second rings (6 → 10 species), the anomalously high value in the third ring necessitates a revised explanatory framework. These findings partially align with studies from Fuzhou (Lin et al. 2023), Kozhikode (Gonzaga 2023), and Dhaka (Chowdhury et al. 2021), which consistently report urbanization‐driven declines in butterfly diversity in city cores (Fang et al. 2023), yet the Harbin case further highlights the unique ecological role of moderately urbanized fringe zones.

Approximately 10 butterfly species were restricted to single parks, displaying distinct spatial and habitat preferences. These specialists clustered in large parks (e.g., TYD, QLP, ZGTY) within the third‐ and fourth‐ring fringe zones, sharing three key traits: (Citaristi 2022) larger area, providing a foundation for habitat heterogeneity and plant diversity; (2) retention of natural vegetation patches (e.g., wetlands in TYD, grasslands in SFT) with abundant herbaceous and flowering plants; and (3) significantly lower management intensity compared to inner‐ring parks. Although exceptions exist (e.g., small parks like SFT), the overall pattern underscores the refuge function of large, naturalized parks in sustaining high richness within highly urbanized areas.

The Harbin case emphasizes the critical role of green space planning: large parks like TYD and QLP preserve natural habitats (e.g., wetlands, herbaceous communities), offering refuges for specialists such as *Aglais urticae* and *Coenonympha amaryllis*. Unlike Beijing's forest‐focused strategy (Han et al. 2021), Harbin's specialists (e.g., *Polyommatus icarus* in SFT's grasslands) exhibit stronger reliance on herbaceous habitats, suggesting northern cities should prioritize vegetation diversity over monocultural greening. Current outer fourth‐ring parks remain constrained by limited numbers and small sizes, necessitating green corridor development to enhance habitat connectivity.

### 4.2 Environmental Factors Influencing Butterfly Species Richness

The results demonstrate that internal park resources and the spatial heterogeneity of the surrounding matrix jointly exert significant influences on butterfly community structure and composition. Specifically, park‐specific characteristics (e.g., internal resources) critically determine butterfly survival and species assembly, while the composition and spatial configuration of adjacent landscapes further shape community dynamics.

#### 4.2.1 The Effect of Park Resources

Variables related to plant resources significantly influenced butterfly communities. Optimal models identified flowering plant richness as a key predictor for butterfly richness and abundance, while overall plant richness drove diversity. These findings align with studies in South Korean Buddhist temple forests and Beijing, where nectar and host plants are critical for butterfly diversity and population conservation (Kim et al. 2012; Han et al. 2022). Research in La Mancha, Mexico (Martínez‐Adriano et al. 2018) emphasized butterflies' strong reliance on flowering plants for foraging, while a Beijing study (Han et al. 2021) highlighted habitat features' importance for butterfly survival and reproduction, noting butterfly richness's sensitivity to overall plant richness in urban parks. This sensitivity likely stems from flowering plants providing nectar and pollen, the primary food sources for adult butterflies (Andow 2023; Hebberecht et al. 2022). The diversity and coverage of flowering plants substantially affect the richness and diversity of visiting arthropods, including butterflies (Hamon et al. 2024; Majewska and Altizer 2020; Williams et al. 2015; Blaauw and Isaacs 2014). Furthermore, flowering plants enhance habitat attractiveness and connectivity, aiding conservation efforts and reducing extinction risks (Milla et al. 2022; Kolkman et al. 2022). The uniqueness of the Harbin case lies in revealing the extreme dependence of butterflies on resource availability in cold‐climate cities.

Park attribute variables similarly impacted butterfly communities. Butterfly species richness negatively correlated with the perimeter–area ratio, which was included in the optimal predictive model. This relationship may arise from intensified edge effects in geometrically complex parks, increasing energy and material exchange rates with surrounding environments (Collinge et al. 2003). In highly urbanized landscapes, irregularly shaped parks become more vulnerable to ambient influences such as light pollution, noise pollution, air pollution, and human activities. Additionally, heightened edge effects reduce core habitat areas, diminishing parks' capacity to protect species and exposing butterflies to external stressors. Some studies reported no significant relationship between butterfly richness and perimeter–area ratio (Han et al. 2021; Di Mauro et al. 2007). Distance from the city center consistently exhibited positive effects on richness, abundance, and diversity, indicating that remoteness from urban cores systematically alleviates urbanization pressures. This phenomenon may be driven by attenuated human activity gradients reducing behavioral interference, weakened heat island effects creating suitable microclimates for stenothermic species like Coenonympha amaryllis, and enhanced connectivity with natural patches in suburban landscapes facilitating species dispersal.

#### 4.2.2 The Effect of Surrounding Landscape

This study, through multi‐model optimization, confirmed that in terms of landscape composition, the proportion of built‐up land serves as the core inhibitory factor for butterfly richness, consistent with findings from Beijing (Han et al. 2021). In Melbourne, increased impervious surface coverage has been shown to reduce butterfly richness and abundance. Urbanization‐driven substitution of natural habitats with impervious surfaces directly diminishes suitable butterfly habitats (Fahrig 2003). Built‐up patches physically and functionally isolate butterfly populations, leading to genetic isolation and disrupted gene flow among local populations (Threlfall et al. 2017). Conversely, natural land‐use types such as forests, croplands, and grasslands positively influence butterfly communities, aligning with studies by Shayna Maskell (Rija 2022), Pendl et al. (2022), and Jain et al. (2017). The urban gradient significantly impacts butterfly populations, with this relationship closely tied to the extent of surrounding natural land cover. Higher natural land cover can mitigate urbanization‐induced declines in butterfly diversity (Blair 2001). Among natural habitats, grassland patches emerge as a critical predictor of butterfly diversity. Unlike forests and croplands, grasslands support generalist species (e.g., *Pieris rapae* ) through open flight corridors and ephemeral nectar resources (e.g., *Trifolium* spp.), a function particularly vital in northern cities where short growing seasons force butterflies to rely on rapid resource turnover.

The regulation of urban butterfly communities by landscape configuration exhibits multi‐dimensional scale dependence. Number of patches (NP) acts as a key predictor of butterfly abundance, validating the moderate fragmentation hypothesis: in urban matrices dominated by built‐up areas, moderate increases in natural patch numbers facilitate “stepping‐stone” networks that reduce dispersal barriers for generalist species and sustain population dynamics through resource complementarity (Tscharntke et al. 2012; Öckinger et al. 2009). However, threshold effects of fragmentation cannot be ignored—excessive patch numbers risk fragmenting core habitats, particularly threatening specialist species reliant on continuous habitats (Fahrig 2003). The strong positive correlation between patch richness (PR) and diversity further reveals that habitat heterogeneity promotes species coexistence via niche differentiation (e.g., functional complementarity between shaded forests and open grasslands), challenging the ecological efficacy of homogeneous greening strategies (Tews et al. 2004; Dennis et al. 2006). The scale‐dependent effects of mean Euclidean nearest neighbor distance (ENN_MN) highlight the complexity of connectivity management: while moderate patch dispersion at small scales may enhance resource‐use efficiency for generalists, large‐scale habitat isolation disrupts gene flow in specialists, triggering local population declines (Hanski 1998). This paradox necessitates spatially differentiated strategies—enhancing landscape heterogeneity through microhabitats in built‐up areas while constructing corridor networks between ecological nodes to mitigate dispersal barriers (Threlfall et al. 2017). Urban butterfly conservation must thus transcend the “fragmented versus continuous” dichotomy, adopting multi‐scale habitat designs to synergistically enhance generalist abundance and specialist diversity.

This study reveals that butterfly community distributions in Harbin are most sensitive to landscape heterogeneity within 100–200 m buffers around parks (lowest AICc values). This likely reflects how highly fragmented urban matrices compress effective ecological processes into localized ranges (< 200 m). Even species theoretically capable of long‐distance dispersal (e.g., *Pieris rapae* ) may experience constrained migration due to building barriers and resource fragmentation, limiting movements to short distances.

### 4.3 Conservation Implications

The richness of flowering plants and overall plant diversity are key factors influencing butterfly species richness. To enhance the diversity of butterfly populations and their breeding habitats, urban park plant resource management should prioritize increasing plant diversity, particularly the variety and abundance of flowering plants. Through rational land allocation and botanical design, park planners and managers can create green spaces conducive to butterfly habitation, thereby supporting the survival and reproduction of butterfly species (Wang et al. 2020). In accordance with the concept of “Ecological Civilization,” urban parks should reserve a certain proportion of native or indigenous plants, designated as unmanaged natural green spaces (Wang et al. 2020). These areas, with their early‐successional plants, positively impact butterflies, often supporting higher plant diversity and ecological quality, making them ideal habitats for butterflies (Sing et al. 2016; Swanson et al. 2011).

Urban parks should not only focus on internal plant resource management but also consider the surrounding landscape, as it profoundly affects butterfly community diversity. Land use types such as forests, farmland, and grasslands have significant positive impacts on butterfly communities, with forest vegetation being particularly critical for butterflies. Therefore, increasing the coverage of these natural landscapes around cities to form green belts or ecological corridors, connecting parks with adjacent natural areas, is essential. Forest coverage typically exhibits the highest species diversity, which not only benefits butterfly habitats but also promotes the overall conservation of urban biodiversity (Pei et al. 2018; Huang et al. 2010; Warren‐Thomas et al. 2014). In Harbin, urban built‐up areas occupy substantial land, and associated urbanization variables negatively affect butterfly species. In planning, cities should refer to the “Kunming‐Montreal Global Biodiversity Framework” and adopt sustainable measures, such as increasing the area, quality, and connectivity of green and blue spaces in urban and densely populated areas. Additionally, to mitigate the negative impacts of urban construction land on butterflies, utilizing river corridors traversing cities has emerged as a viable strategy. Urban river corridors can effectively enhance habitat connectivity, while riparian vegetation provides critical habitat resources for butterflies and other pollinators, further improving butterfly species diversity and abundance (Ancillotto et al. 2024; Zhang et al. 2022).

This study demonstrates that spatial heterogeneity at the 100–200 m scale is most sensitive to changes in butterfly species. Therefore, urban and park planning should account for spatial variations at this scale, particularly in transitional zones between urban built‐up areas, green spaces, roads, and green belts. This will help strengthen the connectivity of butterfly habitats and further promote species diversity.

### 4.4 Limitations and Prospects

Due to limitations in manpower and time, this study may not have fully covered green spaces located farther from urban areas. However, the data collected is still sufficient to reflect the ecological characteristics of the study area. Future research could address this challenge by increasing manpower and resource investment, expanding the sample size, and improving data quality.

Although this study has certain limitations, these issues have not significantly undermined its credibility. On the contrary, they provide guidance for future improvements and contribute to a deeper understanding of butterfly species richness and distribution patterns. We believe that the findings still offer valuable scientific support for ecological conservation and management, and can serve as a reference for developing more effective conservation strategies.

## 5 Conclusions

This study utilized Generalized Linear Mixed Models (GLMMs) to analyze the effects of spatial heterogeneity at different scales on the structure and composition of butterfly communities. The results indicate that spatial heterogeneity metrics play a crucial role in predicting butterfly communities. Specifically, at the 100‐ and 200‐m scales, butterfly populations exhibited strong responses to spatial heterogeneity in the surrounding landscape. This finding deepens our understanding of ecological patterns and distributions of butterfly populations in urban landscapes. The study revealed that by incorporating spatial heterogeneity metrics into the model, its explanatory power and predictive accuracy significantly improved compared to models relying solely on internal park resources. This highlights the combined influence of park resources and surrounding landscape characteristics on butterfly species richness, emphasizing the potential impacts of both the urban matrix and internal resources on butterfly populations. Therefore, strategies for conserving and enhancing butterfly diversity should simultaneously consider habitat resources within parks and the spatial heterogeneity of the surrounding landscape. Additionally, the study demonstrated that plant resource characteristics, such as flowering plants, are critical for protecting and promoting butterfly populations. By strategically configuring plant resources (especially flowering plants) and maintaining herbaceous vegetation, the habitat space for butterflies and other organisms can be effectively conserved, thereby enhancing the health and sustainability of urban green ecosystems.

## Author Contributions

**Kai Wang:** conceptualization (lead), data curation (lead), formal analysis (lead), investigation (lead), methodology (lead), resources (lead), software (lead), validation (lead), visualization (lead), writing – original draft (lead), writing – review and editing (lead). **Yuxin Qi:** supervision (equal), writing – review and editing (equal). **Yuandong Hu:** funding acquisition (equal), project administration (equal), supervision (equal), writing – review and editing (equal). **Dan Han:** funding acquisition (equal), project administration (equal), supervision (equal), writing – review and editing (equal).

## Conflicts of Interest

The authors declare no conflicts of interest.

## Supporting information


**Appendix S1.** Urban butterfly species abundance and relative frequency in Harbin parks.

Appendix S2.

Data S1.

## Data Availability

The data that supports the findings of this study are available in the Appendix S2 of this article.
