# Supplementary material for: Distribution and Influences on Butterfly Diversity in Urban Park Green Spaces: A Case Study of Harbin, China
Source: Ecol Evol. 2025 Jun 12;15(6):e71554. doi: 10.1002/ece3.71554 (PMC12162359; doi:10.1002/ece3.71554)
Supplement: Supplementary file 1 — Appendix S1. Urban butterfly species abundance and relative frequency in Harbin parks. [file ECE3-15-e71554-s003.docx]

**Appendix A.** Urban Butterfly Species Abundance and Relative Frequency in Harbin Parks

| Familia | Subfamilia | Species Name (Latin) | Number of Individuals | Relative Abundance (%) |
| --- | --- | --- | --- | --- |
| Lycaenidae | Lycaeninae | *Lycaena dispar* | 13 | 0.1 |
|  | Polyommatinae | *Celastrina argiolus* | 60 | 0.465 |
|  | Polyommatinae | *Everes argiades* | 1845 | 14.323 |
|  | Polyommatinae | *Plebejus argus* | 42 | 0.325 |
|  | Polyommatinae | *Plebejus argyrognomon* | 12 | 0.093 |
|  | Polyommatinae | *Polyommatus icarus* | 3 | 0.023 |
|  | Polyommatinae | *Polyommatus eros* | 90 | 0.697 |
|  | Polyommatinae | *Tongeia filicaudis* | 2 | 0.015 |
|  | Polyommatinae | *Tongeia. sp. Incer* | 4 | 0.031 |
|  | Polyommatinae | *Tongeia fischeri* | 351 | 2.721 |
|  | Theclinae | *Satyrium iyonis* | 4 | 0.031 |
|  | Theclinae | *Satyrium w-album* | 38 | 0.294 |
| Pieridae | Coliadinae | *Colias erate* | 1695 | 13.144 |
|  | Dismorphiinae | *Leptidea amurensis* | 107 | 0.829 |
|  | Pierinae | *Anthocharis scolymus* | 42 | 0.325 |
|  | Pierinae | *Aporia crataegi* | 193 | 1.527 |
|  | Pierinae | *Pieris napi* | 122 | 0.868 |
|  | Pierinae | *Pieris rapae* | 3760 | 29.352 |
|  | Pierinae | *Pieris melete* | 981 | 7.607 |
|  | Pierinae | *Pontia daplidice* | 884 | 6.87 |
| Nymphalidae | Apaturinae | *Apatura ilia* | 29 | 0.224 |
|  | Apaturinae | *Mimathyma nycteis* | 2 | 0.015 |
|  | Heliconiinae | *Argynnis paphia* | 117 | 0.907 |
|  | Heliconiinae | *Argyronome laodice* | 2 | 0.015 |
|  | Heliconiinae | *Clossiana selenis* | 7 | 0.054 |
|  | Heliconiinae | *Fabriciana adippe* | 2 | 0.015 |
|  | Nymphalinae | *Aglais io* | 2 | 0.015 |
|  | Nymphalinae | *Aglais urticae* | 1 | 0.007 |
|  | Nymphalinae | *Araschnia burejana* | 56 | 0.434 |
|  | Nymphalinae | *Polygonia c-album* | 402 | 3.117 |
|  | Nymphalinae | *Polygonia c-aureum* | 1441 | 11.198 |
|  | Nymphalinae | *Vanessa cardui* | 119 | 0.922 |
|  | Nymphalinae | *Vanessa indica* | 242 | 1.876 |
|  | Satyrinae | *Coenonympha oedippus* | 147 | 1.139 |
|  | Satyrinae | *Coenonympha amaryllis* | 2 | 0.015 |
| Papilionidae | Papilioninae | *Papilio maackii* | 26 | 0.093 |
|  | Papilioninae | *Papilio xuthus* | 2 | 0.201 |
| Hesperiidae | Hesperiinae | *Ochlodes venata* | 12 | 0.093 |
