# Supplementary material for: Distribution and Influences on Butterfly Diversity in Urban Park Green Spaces: A Case Study of Harbin, China
Source: Ecol Evol. 2025 Jun 12;15(6):e71554. doi: 10.1002/ece3.71554 (PMC12162359; doi:10.1002/ece3.71554)
Supplement: Supplementary file 2 — Appendix S2. [file ECE3-15-e71554-s001.docx]

**Table S1.** List of 44 sampled urban parks in Harbin. Urban Zones indicates the location of the park (R1 is within the first ring, R2 is within the second ring, R3 is within the third ring, R4 is within the fourth ring, and R5 is outside the fourth ring but at the edge of the fourth ring). Flo is the total number of flowering plant species in all shrub and herb layers within the park sampling points; Plant is the total number of all plant species in the tree, shrub, and herb layers within the park sampling points; PlantS is the Shannon diversity index of plants in the park; Age(year) is the time since the establishment of the park to 2022; Dis(km) is the straight line distance from the center of the park to the center of the city; Area(ha) is the size of the area within the park where surveys can be conducted; and P is the ratio of the area of the park to the size of the city. A is the ratio of the perimeter of the park to its area.

| Urban Zones | Park | Abbreviation | Richness | Abundance | Flo | Plant | PlantS | Age | Dis  (km) | Area  (ha) | P.A |
| --- | --- | --- | --- | --- | --- | --- | --- | --- | --- | --- | --- |
| R1 | Majiagou Lishui Park | MJG | 1 | 1 | 8 | 32 | 0.000 | 7 | 1.2 | 1.474 | 0.054 |
| R1 | Changqing Park | CQP | 0 | 0 | 13 | 25 | 0.000 | 39 | 1.4 | 4.230 | 0.030 |
| R1 | Children's Park | ETP | 5 | 208 | 45 | 69 | 0.254 | 97 | 1.1 | 19.238 | 0.012 |
| R2 | Guli Park | GLP | 3 | 55 | 14 | 38 | 0.246 | 28 | 4.5 | 11.165 | 0.013 |
| R2 | Hongbo Central Park | HBP | 3 | 70 | 38 | 71 | 0.719 | 7 | 4.2 | 10.890 | 0.013 |
| R2 | Jianguo Park | JGP | 2 | 5 | 7 | 28 | 0.500 | 64 | 3.5 | 3.634 | 0.021 |
| R2 | Jingyu Park | JYP | 4 | 41 | 18 | 37 | 1.005 | 105 | 4.1 | 6.032 | 0.017 |
| R2 | Taiping Park | TPP | 6 | 19 | 22 | 38 | 1.310 | 66 | 5.1 | 5.178 | 0.020 |
| R2 | Wenhua Park | WHP | 3 | 45 | 47 | 57 | 0.714 | 64 | 3.2 | 16.739 | 0.012 |
| R2 | Yushan Park | YSP | 2 | 12 | 19 | 44 | 0.257 | 22 | 3.6 | 4.619 | 0.025 |
| R2 | Zhaolin Park | ZLP | 3 | 30 | 51 | 62 | 0.468 | 116 | 2.7 | 9.727 | 0.014 |
| R2 | Xiangjiang Park | XJP | 3 | 76 | 11 | 24 | 0.667 | 4 | 3.2 | 19.726 | 0.010 |
| R3 | Dongzhi Park | DZP | 2 | 68 | 30 | 37 | 0.263 | 1 | 6 | 1.881 | 0.029 |
| R3 | Fazhandadao Park | FZDD | 6 | 241 | 49 | 69 | 1.254 | 3 | 8.1 | 8.238 | 0.018 |
| R3 | Feicui Park | FCP | 4 | 18 | 27 | 41 | 1.149 | 7 | 8.5 | 2.065 | 0.032 |
| R3 | Qunli Health Ecological Park | QLJK | 8 | 177 | 90 | 77 | 1.614 | 5 | 9.9 | 22.514 | 0.014 |
| R3 | Shangzhi Park | SZP | 3 | 33 | 23 | 40 | 0.690 | 64 | 4.6 | 9.384 | 0.014 |
| R3 | Xianglin Park | XLP | 13 | 315 | 69 | 72 | 1.665 | 4 | 9.6 | 20.827 | 0.010 |
| R3 | Zhongguo Pavilion Park | ZGP | 13 | 1321 | 95 | 120 | 1.980 | 8 | 7.2 | 56.330 | 0.008 |
| R3 | Northeast Forestry University Woodland | NEFU | 17 | 530 | 54 | 88 | 2.069 | 70 | 4.1 | 41.995 | 0.010 |
| R3 | Sun Island Scenic Area | TYD | 22 | 898 | 145 | 131 | 2.316 | 58 | 5.4 | 210.944 | 0.003 |
| R3 | Sports Park | TYP | 7 | 169 | 51 | 78 | 1.242 | 13 | 7.8 | 30.678 | 0.007 |
| R3 | Qunli Bund Wetland Park | QLP | 13 | 2707 | 75 | 99 | 1.487 | 6 | 7.1 | 174.972 | 0.009 |
| R3 | Dingxiang Park | DXP | 8 | 288 | 54 | 84 | 0.932 | 13 | 6.1 | 48.094 | 0.008 |
| R3 | Forest Botanical Park | SLP | 15 | 476 | 181 | 174 | 1.921 | 64 | 6.1 | 117.467 | 0.006 |
| R3 | Laodong Park | LDP | 11 | 163 | 49 | 73 | 2.034 | 5 | 6.7 | 58.722 | 0.006 |
| R4 | Beidahuang Agricultural Park | BDH | 10 | 214 | 73 | 75 | 1.337 | 17 | 9 | 22.653 | 0.011 |
| R4 | Dingxiang Keji Park | DXKJ | 10 | 486 | 45 | 79 | 1.698 | 13 | 10.2 | 18.713 | 0.010 |
| R4 | Guanghan Park | GHP | 7 | 87 | 37 | 79 | 1.303 | 17 | 11.8 | 10.255 | 0.014 |
| R4 | Minsheng Park | MSP | 5 | 64 | 20 | 40 | 1.031 | 13 | 11.1 | 6.736 | 0.018 |
| R4 | Runfu Park | RFP | 6 | 70 | 37 | 58 | 1.596 | 12 | 9.8 | 5.078 | 0.022 |
| R4 | Sifangtai Park | SFT | 10 | 242 | 35 | 39 | 1.927 | 3 | 9.5 | 5.092 | 0.027 |
| R4 | Xinqu Zhongxin Park | XQZX | 9 | 468 | 73 | 88 | 1.171 | 5 | 11.4 | 24.903 | 0.011 |
| R4 | Zhiqing Park | ZQP | 20 | 779 | 78 | 111 | 1.922 | 13 | 12 | 29.931 | 0.014 |
| R5 | Bihai Park | BHP | 11 | 393 | 53 | 55 | 1.809 | 3 | 15.8 | 13.776 | 0.011 |
| R5 | Cuiliu Park | CLP | 12 | 614 | 76 | 79 | 1.209 | 7 | 18.7 | 16.832 | 0.012 |
| R5 | Fujia Xintiandi Park | FJP | 11 | 238 | 60 | 69 | 1.245 | 7 | 18.2 | 5.092 | 0.021 |
| R5 | Jianan Park | JAP | 7 | 77 | 46 | 63 | 1.610 | 7 | 18.9 | 8.483 | 0.015 |
| R5 | Pingfang Park | PFP | 5 | 67 | 60 | 71 | 1.024 | 64 | 18 | 20.037 | 0.010 |
| R5 | Qingnian Park | QNP | 4 | 16 | 25 | 38 | 1.342 | 12 | 16.2 | 2.654 | 0.035 |
| R5 | Tianchi Dingxiang Park | TCP | 8 | 153 | 37 | 65 | 1.585 | 13 | 14.7 | 15.644 | 0.010 |
| R5 | Xiaohong Park | XHP | 15 | 580 | 63 | 65 | 1.723 | 12 | 17.3 | 9.411 | 0.019 |
| R5 | Xinqu Hangtian Park | XQHT | 10 | 119 | 54 | 58 | 1.773 | 3 | 14.4 | 12.941 | 0.012 |
| R5 | Zijing Park | ZJP | 8 | 216 | 37 | 53 | 1.559 | 5 | 16.6 | 5.771 | 0.016 |

**Table S2. Distribution of Butterfly Species in Urban Parks Across Ring Road Zones (R1-R5) in Harbin.** **R1 is the region inside the first ring, R2 is the region inside the second ring, R3 is the region inside the third ring, R4 is the region inside the fourth ring, and R5 is the region outside the fourth ring.**

|  | **CQP**  **(R1)** | **ETP**  **(R1)** | **MJG**  **(R1)** | **GLP**  **(R2)** | **HBP**  **(R2)** | **JGP**  **(R2)** | **JYP**  **(R2)** | **TPP**  **(R2)** | **WHP**  **(R2)** | **XJP**  **(R2)** | **YSP**  **(R2)** | **ZLP**  **(R2)** | **DXP**  **(R3)** | **DZP**  **(R3)** | **FCP**  **(R3)** | **FZDD**  **(R3)** | **LDP**  **(R3)** | **NEFU**  **(R3)** | **QLJK**  **(R3)** | **QLP**  **(R3)** | **SLP**  **(R3)** | **SZP**  **(R3)** |
| --- | --- | --- | --- | --- | --- | --- | --- | --- | --- | --- | --- | --- | --- | --- | --- | --- | --- | --- | --- | --- | --- | --- |
| *Aglais io* |  |  |  |  |  |  |  |  |  |  |  |  |  |  |  |  |  | 1 |  |  |  |  |
| *Aglais urticae* |  |  |  |  |  |  |  |  |  |  |  |  |  |  |  |  |  |  |  |  |  |  |
| *Anthocharis scolymus* |  |  |  |  |  |  |  |  |  |  |  |  | 1 |  |  |  |  | 11 |  |  | 1 |  |
| *Apatura ilia* |  |  |  |  |  |  |  |  |  |  |  |  |  |  |  |  | 6 |  |  |  |  |  |
| *Aporia crataegi* |  |  |  |  |  |  | 11 | 1 |  |  |  |  |  |  |  | 2 |  |  |  |  | 6 |  |
| *Araschnia burejana* |  |  |  |  |  |  |  |  |  |  |  |  |  |  |  |  |  | 15 |  |  |  |  |
| *Argynnis paphia* |  |  |  |  |  |  |  |  |  |  | 1 |  |  |  |  |  |  | 9 |  |  | 21 |  |
| *Argyronome laodice* |  |  |  |  |  |  |  |  |  |  |  |  |  |  |  |  |  |  |  |  |  |  |
| *Celastrina argiolus* |  |  |  |  |  |  |  |  |  |  |  |  | 3 |  |  |  |  |  |  |  |  |  |
| *Clossiana selenis* |  |  |  |  |  |  |  |  |  |  |  |  |  |  |  |  |  |  |  |  |  |  |
| *Coenonympha amaryllis* |  |  |  |  |  |  |  |  |  |  |  |  |  |  |  |  |  |  |  | 2 |  |  |
| *Coenonympha oedippus* |  |  |  |  |  |  |  |  |  |  |  |  |  |  |  |  |  |  |  |  |  |  |
| *Colias erate* |  |  |  |  | 7 |  |  | 3 |  | 5 |  |  | 13 |  |  | 59 | 2 | 60 | 39 | 514 | 29 | 2 |
| *Everes argiades* |  | 2 |  |  |  |  | 2 |  | 15 | 12 |  |  | 29 |  | 2 | 60 | 3 | 10 | 30 | 263 | 44 | 6 |
| *Fabriciana adippe* |  |  |  |  |  |  |  |  |  |  |  |  |  |  |  |  |  |  |  |  |  |  |
| *Leptidea amurensis* |  |  |  |  |  |  |  | 1 |  |  |  |  |  |  |  |  |  |  |  |  |  |  |
| *Lycaena dispar* |  |  |  |  |  |  |  |  |  |  |  |  |  |  |  |  |  |  |  | 6 |  |  |
| *Mimathyma nycteis* |  |  |  |  |  |  |  |  |  |  |  |  |  |  |  |  |  | 2 |  |  |  |  |
| *Ochlodes venata* |  |  |  |  |  |  |  |  |  |  |  |  |  |  |  |  |  |  |  |  |  |  |
| *Papilio maackii* |  |  |  |  |  |  |  |  |  |  |  |  |  |  |  |  | 6 | 4 |  |  | 11 |  |
| *Papilio xuthus* |  |  |  |  |  |  |  |  |  |  |  |  |  |  |  |  |  | 2 |  |  |  |  |
| *Pieris melete* |  |  |  |  |  |  |  |  |  |  |  |  | 7 |  |  |  | 29 | 161 |  | 1 | 147 |  |
| *Pieris napi* |  |  |  |  |  |  |  |  |  |  |  |  |  |  |  |  |  | 44 |  |  | 14 |  |
| *Pieris rapae* |  | 198 | 1 |  | 53 | 4 | 27 | 11 | 32 | 59 | 13 | 26 | 219 | 63 | 10 | 108 | 40 | 92 | 72 | 1020 | 135 | 25 |
| *Plebejus argus* |  |  |  | 52 |  |  |  |  |  |  |  |  |  |  |  |  |  |  |  | 1 |  |  |
| *Plebejus argyrognomon* |  |  |  |  |  |  |  |  |  |  |  |  |  |  |  |  |  |  |  | 1 |  |  |
| *polygonia c-album* |  | 1 |  |  |  |  |  | 2 |  |  |  |  |  |  |  |  | 20 | 86 |  |  | 8 |  |
| *Polygonia c-aureum* |  | 3 |  | 2 | 10 | 1 | 4 | 1 | 1 |  |  | 1 | 11 | 5 | 4 | 11 | 16 | 20 | 18 | 122 | 50 |  |
| *Polyommatus eros* |  |  |  |  |  |  |  |  |  |  |  |  |  |  |  |  |  |  |  | 19 | 1 |  |
| *Polyommatus icarus* |  |  |  |  |  |  |  |  |  |  |  |  |  |  |  |  |  |  |  |  |  |  |
| *Pontia daplidice* |  |  |  |  |  |  |  |  |  |  |  |  | 5 |  |  |  | 1 | 4 | 2 | 751 | 3 |  |
| *Satyrium iyonis* |  |  |  |  |  |  |  |  |  |  |  |  |  |  |  |  |  |  |  |  |  |  |
| *Satyrium w-album* |  |  |  |  |  |  |  |  |  |  |  |  |  |  |  |  | 28 |  |  |  |  |  |
| *Tongeia filicaudis* |  |  |  |  |  |  |  |  |  |  |  |  |  |  |  |  |  |  |  |  |  |  |
| *Tongeia fischeri* |  |  |  |  |  |  |  |  |  |  |  |  |  |  |  |  |  |  | 7 | 3 |  |  |
| *Tongeia. sp. Incer* |  |  |  |  |  |  |  |  |  |  |  |  |  |  |  |  |  |  |  |  |  |  |
| *Vanessa cardui* |  | 4 |  |  |  |  |  |  |  |  |  |  |  |  | 2 |  | 12 | 7 | 2 |  | 4 |  |
| *Vanessa indica* |  |  |  | 1 |  |  |  |  |  |  |  | 3 |  |  |  | 1 |  | 2 | 10 | 5 | 2 |  |

|  | **TYD**  **(R3)** | **TYP**  **(R3)** | **XLP**  **(R3)** | **ZGTY**  **(R3)** | **BDH**  **(R4)** | **DXKJ**  **(R4)** | **GHP**  **(R4)** | **RFP**  **(R4)** | **SFT**  **(R4)** | **XQZX**  **(R4)** | **ZQP**  **(R4)** | **BHP**  **(5R)** | **CLP**  **(5R)** | **FJP**  **(5R)** | **JAP**  **(5R)** | **PFP**  **(5R)** | **QNP**  **(5R)** | **TCP**  **(5R)** | **XHP**  **(5R)** | **XQHT**  **(5R)** | **ZJP**  **(5R)** | **MSP**  **(R4)** |
| --- | --- | --- | --- | --- | --- | --- | --- | --- | --- | --- | --- | --- | --- | --- | --- | --- | --- | --- | --- | --- | --- | --- |
| *Aglais io* |  |  |  |  |  |  |  |  |  |  | 1 |  |  |  |  |  |  |  |  |  |  |  |
| *Aglais urticae* | 1 |  |  |  |  |  |  |  |  |  |  |  |  |  |  |  |  |  |  |  |  |  |
| *Anthocharis scolymus* | 22 |  |  |  |  |  |  |  |  |  |  |  | 7 |  |  |  |  |  |  |  |  |  |
| *Apatura ilia* |  |  |  |  |  |  |  |  |  |  | 20 |  |  |  |  |  |  |  | 1 |  |  | 2 |
| *Aporia crataegi* |  |  | 2 | 41 | 2 | 23 | 1 |  |  |  | 1 |  | 28 | 5 | 25 | 23 |  | 2 | 24 |  |  |  |
| *Araschnia burejana* | 37 |  |  |  |  |  |  |  |  |  | 2 |  | 2 |  |  |  |  |  |  |  |  |  |
| *Argynnis paphia* | 12 |  |  |  |  |  | 14 |  |  |  | 8 |  | 34 | 3 | 2 | 1 | 3 |  | 9 |  |  |  |
| *Argyronome laodice* |  |  |  | 2 |  |  |  |  |  |  |  |  |  |  |  |  |  |  |  |  |  |  |
| *Celastrina argiolus* | 1 |  | 1 | 34 |  |  |  |  |  |  | 21 |  |  |  |  |  |  |  |  |  |  |  |
| *Clossiana selenis* | 2 |  | 3 | 2 |  |  |  |  |  |  |  |  |  |  |  |  |  |  |  |  |  |  |
| *Coenonympha amaryllis* |  |  |  |  |  |  |  |  |  |  |  |  |  |  |  |  |  |  |  |  |  |  |
| *Coenonympha oedippus* | 146 |  |  |  |  |  |  |  |  |  | 1 |  |  |  |  |  |  |  |  |  |  |  |
| *Colias erate* | 86 | 23 | 9 | 175 | 9 | 6 |  | 6 | 52 |  | 317 | 106 | 3 | 15 | 2 |  |  | 16 | 53 | 12 | 72 |  |
| *Everes argiades* | 109 | 10 | 120 | 117 | 72 | 24 | 9 | 14 | 60 | 34 | 147 | 50 | 28 | 154 | 2 |  | 3 | 13 | 284 | 46 | 27 | 46 |
| *Fabriciana adippe* |  |  |  | 2 |  |  |  |  |  |  |  |  |  |  |  |  |  |  |  |  |  |  |
| *Leptidea amurensis* | 8 |  | 73 |  | 1 | 12 |  |  |  |  | 5 | 3 |  |  |  |  |  |  | 4 |  |  |  |
| *Lycaena dispar* | 6 |  |  |  |  |  |  |  |  |  |  |  |  |  |  |  |  |  | 1 |  |  |  |
| *Mimathyma nycteis* |  |  |  |  |  |  |  |  |  |  |  |  |  |  |  |  |  |  |  |  |  |  |
| *Ochlodes venata* | 1 |  | 2 |  |  |  |  |  |  |  | 4 |  |  |  |  |  |  |  |  | 5 |  |  |
| *Papilio maackii* | 1 |  |  |  |  |  |  |  |  |  | 4 |  |  |  |  |  |  |  |  |  |  |  |
| *Papilio xuthus* |  |  |  |  |  |  |  |  |  |  |  |  |  |  |  |  |  |  |  |  |  |  |
| *Pieris melete* | 123 |  | 5 | 3 | 2 |  |  |  |  | 5 | 27 |  | 431 | 2 | 17 | 1 | 6 | 5 | 5 | 3 | 1 |  |
| *Pieris napi* | 46 |  |  |  |  |  |  |  |  |  |  |  | 8 |  |  |  |  |  |  |  |  |  |
| *Pieris rapae* | 84 | 102 | 31 | 379 | 97 | 197 | 8 | 26 | 55 | 43 | 54 | 59 | 14 | 39 | 13 | 39 | 4 | 66 | 101 | 29 | 75 | 10 |
| *Plebejus argus* |  |  |  |  |  |  |  |  | 19 |  |  | 7 |  |  |  |  |  |  | 15 |  |  |  |
| *Plebejus argyrognomon* |  |  |  |  |  |  |  |  | 6 | 4 |  | 1 |  |  |  |  |  |  |  |  |  |  |
| *polygonia c-album* | 2 |  |  | 193 |  | 61 |  |  |  |  | 9 |  |  | 2 |  |  |  |  |  | 2 | 12 | 4 |
| *Polygonia c-aureum* | 181 | 23 | 63 | 218 | 26 | 125 | 52 | 13 | 26 | 44 | 106 | 114 | 48 | 13 | 16 | 6 |  | 38 | 14 | 9 | 23 | 6 |
| *Polyommatus eros* | 9 | 1 |  |  |  |  |  |  | 5 |  | 28 | 10 |  |  |  |  |  |  | 17 |  |  |  |
| *Polyommatus icarus* |  |  |  |  |  |  |  |  | 3 |  |  |  |  |  |  |  |  |  |  |  |  |  |
| *Pontia daplidice* | 6 | 8 | 3 |  | 3 |  |  | 8 | 10 | 9 | 4 | 16 |  | 1 |  |  |  |  | 42 | 5 | 5 |  |
| *Satyrium iyonis* |  |  | 4 |  |  |  |  |  |  |  |  |  |  |  |  |  |  |  |  |  |  |  |
| *Satyrium w-album* | 10 |  |  |  |  |  |  |  |  |  |  |  |  |  |  |  |  |  |  |  |  |  |
| *Tongeia filicaudis* |  |  |  |  |  | 2 |  |  |  |  |  |  |  |  |  |  |  |  |  |  |  |  |
| *Tongeia fischeri* | 5 |  |  |  |  |  |  |  | 10 | 317 |  |  | 9 |  |  |  |  |  |  |  |  |  |
| *Tongeia. sp. Incer* |  |  |  |  |  |  |  |  |  | 4 |  |  |  |  |  |  |  |  |  |  |  |  |
| *Vanessa cardui* |  | 2 |  | 37 | 1 | 17 | 2 |  |  |  | 17 | 2 |  | 3 |  |  |  | 4 | 2 | 1 |  |  |
| *Vanessa indica* |  |  | 1 | 118 | 1 | 25 | 3 | 3 |  | 8 | 3 | 25 | 2 | 2 |  |  |  | 9 | 8 | 7 | 3 |  |

**Table S3. AICc-Based Multi-Scale Optimal Model Selection and Goodness-of-Fit Evaluation for Butterfly Community Metrics**

| Butterfly metric | Range | variable | aicc | R2m | R2c |
| --- | --- | --- | --- | --- | --- |
| Abundance | 0 | Area + Distance + Flo | 1603.9 | 0.429773 | 0.816155 |
| Abundance | 100 | Flo + Distance + A_MN_Grass100 + A_AM_Built100 + X100NP + X100ENN_MN | 1595.2 | 0.531029 | 0.804756 |
| Abundance | 200 | Flo + Distance + X200NP + X200ENN_MN | 1591 | 0.531988 | 0.81134 |
| Abundance | 350 | Flo + Distance + A_AM_Built350 + A_AM_Grass350 +A_AM_Tree350 + X350NP | 1594.7 | 0.537624 | 0.804727 |
| Abundance | 500 | Flo + A_MN_Bare500 +A_AM_Tree500 +X500NP + X500COHESION | 1593.8 | 0.526973 | 0.811321 |
| Abundance | 750 | Flo + PLAND_Tree750 + X750NP + X750SHAPE_MN | 1595.3 | 0.508867 | 0.808205 |
| Abundance | 1000 | Flo + A_MN_Crop1000 + A_MN_Tree1000 + X1000NP | 1594.8 | 0.5121 | 0.807067 |
| Abundance | 1500 | Area + Flo + A_MN_Crop1500 + A_MN_Tree1500 + A_AM_Built1500 + X1500NP | 1592.8 | 0.551572 | 0.81232 |
| Abundance | 2400 | Flo + Age + A_MN_Crop2400 + A_MN_Tree2400 + X2400PR + X2400PRD | 1594.3 | 0.542791 | 0.809101 |
| Diversity | 0 | Distance + Plant | 354.4 | 0.147511 | 0.147511 |
| Diversity | 100 | Distance + Plant | 249.4 | 0.285897 | 0.344243 |
| Diversity | 200 | Plant + Distance + PLAND_Grass200 + A_MN_Built200 +A_AM_Bare200 + X200PR | 239.8 | 0.379501 | 0.388843 |
| Diversity | 350 | Plant + Distance + A_AM_Grass350 | 245.7 | 0.283943 | 0.29211 |
| Diversity | 500 | Plant +A_MN_Bare500 +X500COHESION + X500PR | 242.3 | 0.33318 | 0.343009 |
| Diversity | 750 | Plant + A_AM_Built750 +X750NP + X750SHAPE_MN | 253.7 | 0.246787 | 0.250073 |
| Diversity | 1000 | Plant + X1000ECON_MN | 241.9 | 0.275586 | 0.28419 |
| Diversity | 1500 | Plant + A_MN_Tree1500 + X1500NP | 252.5 | 0.265156 | 0.265156 |
| Diversity | 2400 | Plant + PlantS +PLAND_Crop2400 + PLAND_Tree2400 | 243 | 0.301799 | 0.305344 |
| Richness | 0 | Area + Distance + Flo | 717.1 | 0.420582 | 0.643057 |
| Richness | 100 | P.A + Flo + Distance + PLAND_Built100 | 697.4 | 0.572198 | 0.678125 |
| Richness | 200 | Flo + PlantS + Distance + PLAND_Grass200 + A_AM_Bare200 + X200ENN_MN +X200PR | 705 | 0.542459 | 0.626311 |
| Richness | 350 | Flo + PlantS + A_MN_Built350 + A_AM_Grass350 | 711 | 0.506177 | 0.626731 |
| Richness | 500 | Flo + PlantS + A_MN_Bare500 + X500COHESION + X500PR | 709.4 | 0.497294 | 0.617408 |
| Richness | 750 | Flo + PLAND_Tree750 +A_AM_Bare750 + X750NP | 705.6 | 0.506677 | 0.628284 |
| Richness | 1000 | Flo + PlantS + A_MN_Crop1000 + A_MN_Tree1000 + A_AM_Bare1000 +A_AM_Built1000 + A_AM_Grass1000 +X1000SHAPE_MN + X1000PR | 707.7 | 0.535139 | 0.613794 |
| Richness | 1500 | Flo + A_MN_Tree1500 + A_MN_water1500 + X1500NP | 706.3 | 0.50661 | 0.630132 |
| Richness | 2400 | Flo + PLAND_Crop2400 + X2400PRD | 703.1 | 0.498405 | 0.610259 |


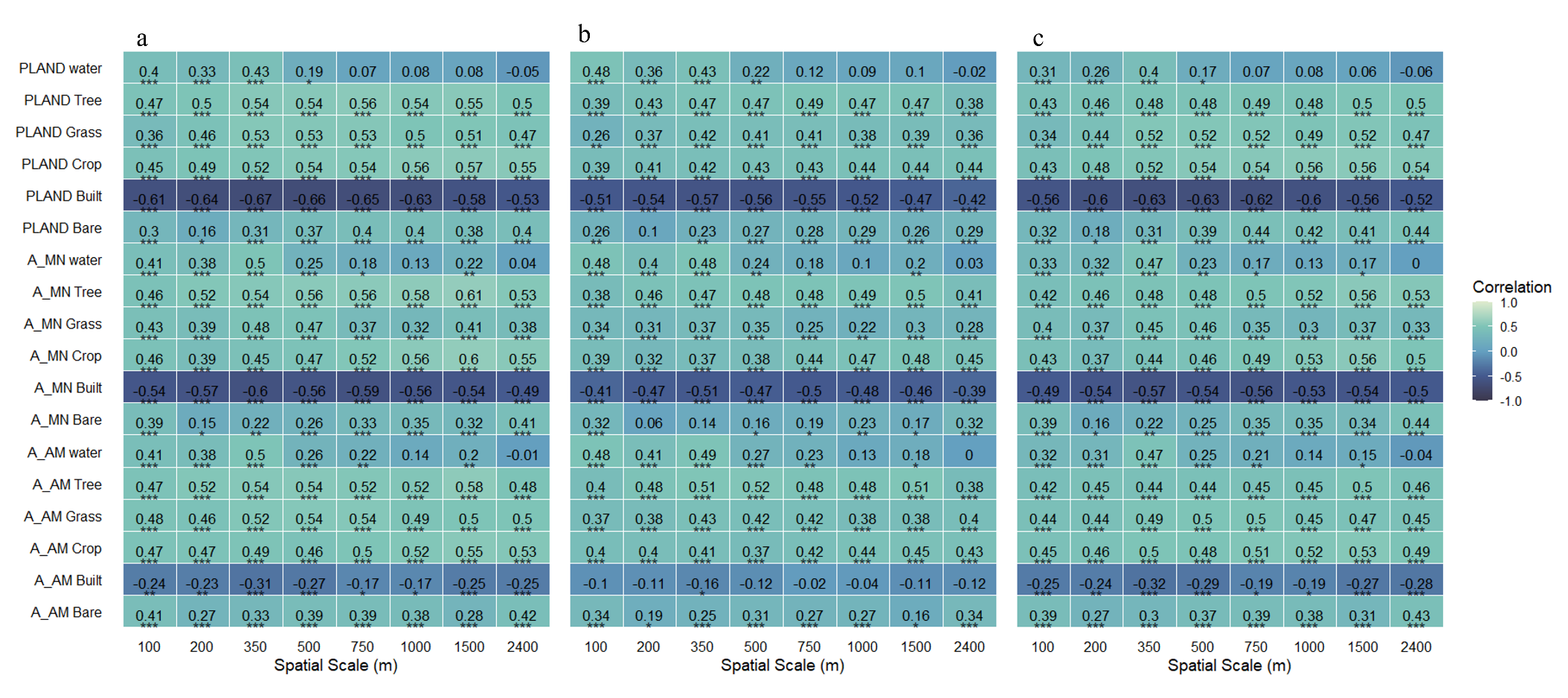


**Figure S1.** Spearman’s correlation coefficient between landscape composition and butterfly richness at different spatial scales. (a) Correlation heatmap between butterfly species richness and landscape indices; (b) Correlation heatmap between butterfly abundance and landscape indices; (c) Correlation heatmap between butterfly Shannon diversity and landscape indices. *** indicates significance at the 0.001 level, ** indicates significance at the 0.01 level, and * indicates significance at the 0.05 level (two-tailed). PLAND represents the percentage of landscape of each land cover type, A_MN represents the mean patch area of each land cover type, and A_AM represents the area-weighted mean patch area of each land cover type.


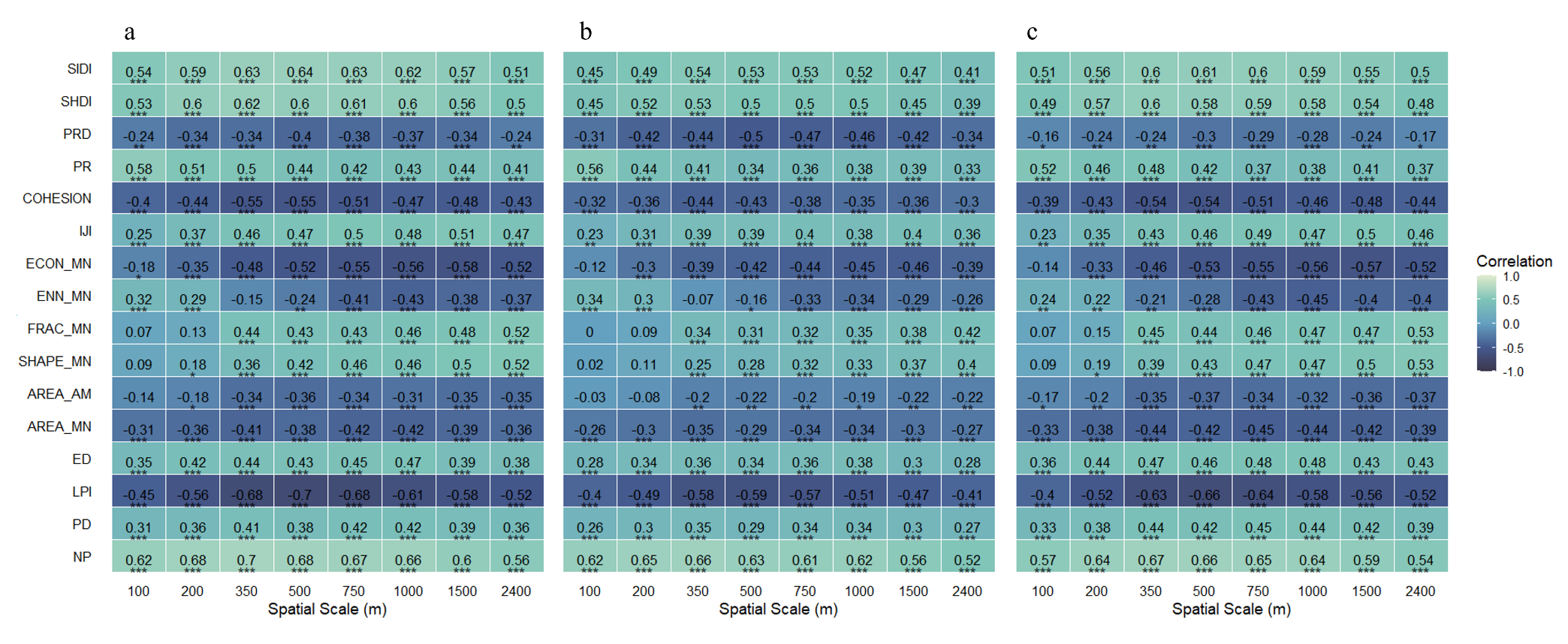


**Figure S2.** Spearman’s correlation coefficients between landscape configurations and butterfly species at different spatial scales. (a) Correlation heatmap between butterfly species richness and landscape indices; (b) Correlation heatmap between butterfly abundance and landscape indices; (c) Correlation heatmap between butterfly Shannon diversity and landscape indices.*** indicates significance at the 0.001 level, ** indicates significance at the 0.01 level, and * indicates significance at the 0.05 level (two-tailed). NP represents the number of patches, PD represents patch density, LPI represents the largest patch index, ED represents edge density, AREA_MN represents mean patch area, AREA_AM represents area-weighted mean patch area, SHAPE_MN represents mean shape index, FRAC_MN represents mean fractal dimension index, ENN_MN represents mean Euclidean nearest neighbor distance, ECON_MN represents mean edge contrast index, IJI represents interspersion juxtaposition index, COHESION represents patch cohesion index, PR represents patch richness, PRD represents patch richness density, SHDI represents Shannon’s diversity index, and SIDI represents Simpson’s diversity index.
